# *PIK3CA* Cooperates with *KRAS* to Promote MYC Activity and Tumorigenesis via the Bromodomain Protein BRD9

**DOI:** 10.3390/cancers11111634

**Published:** 2019-10-24

**Authors:** Catherine M. Bell, Philipp Raffeiner, Jonathan R. Hart, Peter K. Vogt

**Affiliations:** Department of Molecular Medicine, The Scripps Research Institute, 10550 North Torrey Pines Road, La Jolla, CA 92037, USA; philippr@scripps.edu (P.R.); jhart@scripps.edu (J.R.H.); pkvogt@scripps.edu (P.K.V.)

**Keywords:** phosphatidylinositol 3-kinase (PI 3-kinase), GTPase Kras (KRAS), MYC (c-Myc), bromodomain-containing protein 9 (BRD9), oncogene, tumor cell biology, transcriptomics, chromatin remodeling, CRISPR-Cas9, transformation

## Abstract

Tumor formation is generally linked to the acquisition of two or more driver genes that cause normal cells to progress from proliferation to abnormal expansion and malignancy. In order to understand genetic alterations involved in this process, we compared the transcriptomes of an isogenic set of breast epithelial cell lines that are non-transformed or contain a single or double knock-in (DKI) of PIK3CA (H1047R) or KRAS (G12V). Gene set enrichment analysis revealed that DKI cells were enriched over single mutant cells for genes that characterize a MYC target gene signature. This gene signature was mediated in part by the bromodomain-containing protein 9 (BRD9) that was found in the SWI-SNF chromatin-remodeling complex, bound to the MYC super-enhancer locus. Small molecule inhibition of BRD9 reduced MYC transcript levels. Critically, only DKI cells had the capacity for anchorage-independent growth in semi-solid medium, and CRISPR-Cas9 manipulations showed that PIK3CA and BRD9 expression were essential for this phenotype. In contrast, KRAS was necessary for DKI cell migration, and BRD9 overexpression induced the growth of KRAS single mutant cells in semi-solid medium. These results provide new insight into the earliest transforming events driven by oncoprotein cooperation and suggest BRD9 is an important mediator of mutant PIK3CA/KRAS-driven oncogenic transformation.

## 1. Introduction

The tumorigenic potential of an oncogene is defined by its cooperation with other driver mutations that result in complex changes to a cell’s organization [1,2]. *PIK3CA*, the gene encoding p110α, the catalytic subunit of phosphatidylinositol 3-kinase (PI3K), is one of the most frequently mutated genes in human cancer [3]. The hotspot mutation H1047R occurs within the kinase domain of p110α and results in higher plasma membrane recruitment and lipid kinase activity than the wild type protein [4]. Activated PI3K is central to tumor biology by controlling a multitude of cellular activities, including metabolism, genomic stability, cell motility and proliferation [5].

Activating mutations in *PIK3CA* can co-occur with other driver mutations [6]. An example of this is the presence of both mutant *PIK3CA* and mutant *KRAS* (GTPase-encoding oncogene, first identified in the Kirsten rat-sarcoma virus). Activation of either protein alone results in increased signaling through both the PI3K and mitogen-activated protein kinase (MAPK) pathways [7]. Data from human tumor sequencing efforts have shown that KRAS and PIK3CA mutations can co-occur, but their co-occurrence depends upon the disease. Colon cancer shows significant co-occurrence [8], whereas in lung cancer there is mutual exclusivity [9]. Although KRAS mutations are not frequent in breast cancer, they are significantly associated with PIK3CA mutations [10,11,12].

There have been numerous studies analyzing the interactions of RAS and PI3K [13,14,15]. The starting point for the present investigation is a study by Park’s group which established an isogenic set of breast epithelial cell lines that are either wild type with respect to PIK3CA or KRAS or contain a single or double knock-in (DKI) of PIK3CA (H1047R) and/or KRAS (G12V) [16]. These authors discovered that only the DKI cells have the capacity to form tumors in immuno-compromised mice, and these tumors retain single copies of each allele. There is a qualitative difference between single and DKI reflecting the acquisition of overt oncogenicity. This groundbreaking work also documented the importance of a physical KRAS-PI3K interaction in the oncogenicity of DKI cells as well as DKI-specific activation of p90RSK and p70RSK.

The purpose of the current study is a molecular analysis of the interaction of PIK3CA (H1047R) and KRAS (G12V) in MCF-10A cells to identify and elucidate the mediators that are important in the oncogenicity of the DKI MCF-10A. Although this experimental system is not a model for any particular human cancer, it provides instructive and medically relevant insights into the complementing effects of two important oncoproteins. We use next generation sequencing in conjunction with CRISPR-dCas9-KRAB interference (CRISPRi) knock-down of candidate target genes and highlight the bromodomain protein BRD9 as an important facilitator of the MYC target gene signature characteristic of DKI cells and of their oncogenic potency.

## 2. Results

### 2.1. Contribution of Oncogenic PIK3CA and KRAS to Anchorage-Independent Growth, Proliferation and Migration

**Figure 1:**
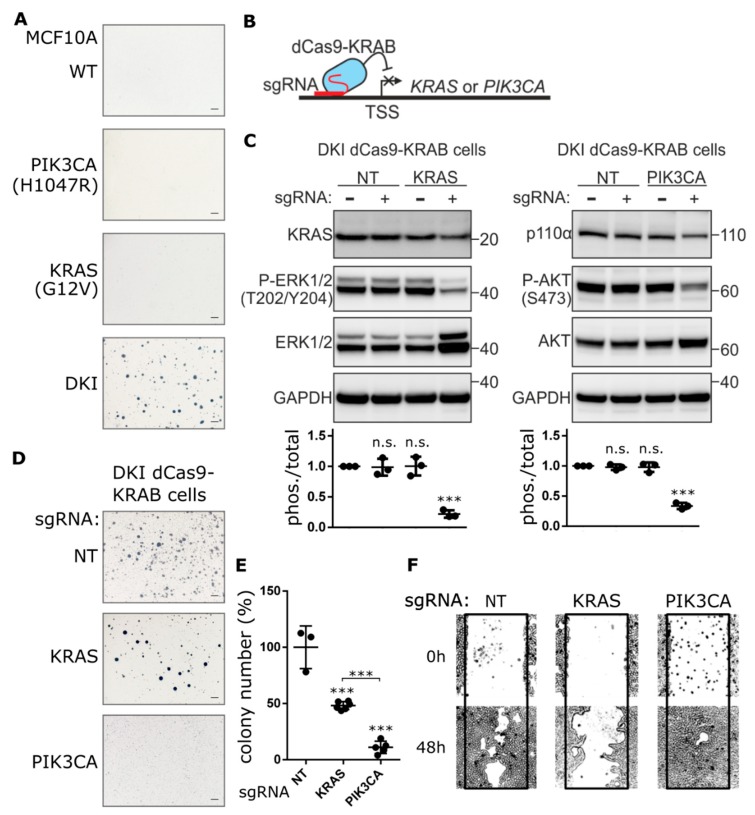
Knockdown of PIK3CA expression blocks anchorage-independent growth, not migration. (**A**) Representative images showing colony formation by MCF-10A cells grown in 0.3% agarose for 15 days. (**B**) Cartoon depicting CRISPRi-mediated gene repression. Single guide RNA (sgRNA) targets nuclease-dead Cas9 (dCas9), fused to the transcriptional repressor KRAB, to the transcription start site (TSS) of the gene of interest, thereby blocking transcription initiation. (**C**) KRAS and p110α protein levels determined by immunoblot after CRISPRi-mediated repression of KRAS or PIK3CA genes in DKI cells. Downregulation of KRAS and PI3K signaling was assessed using phospho-specific antibodies targeting ERK (Thr202/Tyr204) and AKT (Ser473). Densitometric quantification of phospho vs. total blots (lower panels, Mean ± SD). (**D**) Soft agar colony formation after 21 days by DKI cells stably transduced with KRAS or PIK3CA targeting CRISPRi constructs. (**E**) Quantification of soft agar colonies (Mean ± SD). (**F**) Migration of DKI-dCas9-KRAB cells across a scratched wound over 48 h. ***, *p* < 0.001 (unpaired *t*-test); n.s., not significant; NT, non-targeting sgRNA; scale bars = 100 μm.

In order to understand the effects of oncogenic *PIK3CA* in the context of a second driver mutation, we employed a set of MCF-10A cell lines that differ by a single amino acid substitution in PI3K (H1047R) or KRAS (G12V) or that contain both mutations (referred to as DKI) [16]. These mutations occur within the catalytic domains of each gene and lead to constitutive activation and hyperactive downstream signaling through AKT or ERK, respectively (Figure S1A). Individually, the presence of mutant *KRAS* or *PIK3CA* leads to epidermal growth factor (EGF)-independent cell proliferation (Figure S1B), a feature associated with an increased malignant potential [17]. Nevertheless, only DKI cells have the capacity to form tumors in immunocompromised mice [16]. To validate this capacity for tumorigenesis in vitro, we performed anchorage-independent growth assays in semisolid medium containing EGF. In concordance with published data [16], only DKI cells formed colonies in soft agar (Figure 1A). Likewise, only DKI cells were able to grow on plates coated with a nonionic hydrophilic layer (poly-2-hydroxyethyl methacrylate or polyHEMA) (Figure S1B). Furthermore, DKI cells proliferated at a higher rate and migrated across a scratched surface faster than wild type or single mutant cells in EGF-free medium (Figure S1C). These data defined a cell-based model of transformation by oncogene cooperation.

To determine the extent to which *PIK3CA* (H1047R) or *KRAS* (G12V) contribute to this oncogenic phenotype, each gene was inhibited separately in DKI cells by CRISPRi. Anchorage-independent growth, proliferation and migration were used as indirect measures of transformation. Since CRISPR-Cas9-mediated knockout of these genes resulted in significant MCF-10A cell death (Figure S2A), we generated stable MCF-10A DKI cells that expressed catalytically dead Cas9 (dCas9) fused to the transcriptional repressor peptide KRAB (Figure 1B and Figure S2B). Multiple single guide RNAs (sgRNAs) were designed to target the transcriptional start site (TSS) of *PIK3CA* or *KRAS*, and knockdown efficiency was evaluated by RT-qPCR and immunoblot (Figure S2C,D). Guides with the best knockdown efficiency as well as a non-targeting (NT) guide were transduced into DKI-dCas9-KRAB cells for anchorage-independent growth and migration assays. Knockdown of *KRAS* or *PIK3CA* reduced the phosphorylation of ERK1/2 and AKT, respectively (Figure 1C), and significantly decreased DKI cell proliferation (Figure S2E). However, *PIK3CA* knockdown effectively blocked soft agar growth (Figure 1D), resulting in significantly fewer colonies than DKI cells expressing NT or *KRAS* TSS-targeting sgRNA (Figure 1E). Surprisingly, when migration capacity was challenged, the reverse effect was seen: *KRAS* knockdown strongly inhibited DKI cell migration across a scratched wound, while *PIK3CA* knockdown maintained the same migration capacity as control cells expressing the NT sgRNA (Figure 1F).

Several chemical inhibitors of the PI3K pathway exist and are undergoing clinical evaluation, and while an effective KRAS inhibitor remains elusive, there are several effective inhibitors of the MAPK pathway. Chemical inhibition of MAPK (with MEK inhibitor AZD6244) or PI3K (with p110α-specific inhibitor A66) pathways resulted in a dose-dependent decrease in phosphorylation of AKT and ERK1/2 (Figure S3A) as well as MCF-10A cell proliferation (Figure S3B). However, while AZD6244 reduced soft agar colony size, inhibition of PI3K led to a complete loss of soft agar growth (Figure S3C). Although AZD6244-treatment leads to cell-cycle arrest, it has no significant impact on cell viability (Figure S4A,B). Collectively, these findings suggest that oncogenic *PIK3CA* contributes to the ability of DKI cells to grow in an anchorage-independent manner, while oncogenic *KRAS* contributes to the enhanced migration capacity of DKI cells.

### 2.2. KRAS and PIK3CA Oncogenes Cooperate to Generate a Transcriptional Signature Enriched for EMT, Cell Cycle and MYC Target Genes

**Figure 2:**
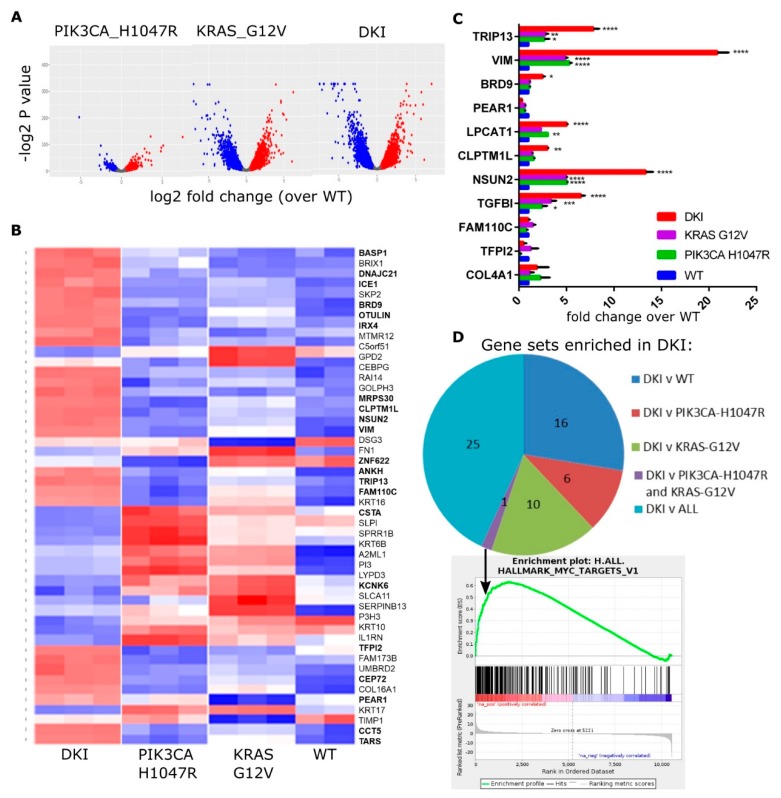
The KRAS and PIK3CA oncogenes cooperate to yield a MYC-target gene signature in DKI cells. (**A**) Volcano plots summarizing gene expression data, mutant vs. WT cells (log2FC vs. *p* value). (**B**) Heat map showing the top 50 upregulated (red) and downregulated (blue) genes in mutant cells compared to WT cells. Columns represent sequence reads from three separate RNA isolates per mutant cell line. (**C**) RT-qPCR data (Mean ± SEM, n = 3) showing mRNA levels of top DE genes in MCF-10A cells as identified by RNAseq. Data analyzed by 2-way ANOVA with Sidak’s multiple comparisons test (*, *p* < 0.05; **, *p* < 0.01; ***, *p* < 0.001). (**D**) Pie chart showing the number of Hallmark gene sets enriched in DKI cells as determined by GSEA of DKI vs. WT or single mutant cells. Bottom panel: Enrichment plot for gene set MYC_TARGETS_V1 generated using the rank ordered DE gene list of DKI vs. all comparisons.

The MCF-10A cell lines each have distinct phenotypic changes that are important for transformation. By utilizing next-generation sequencing, we can define the gene expression changes leading to anchorage-independent growth and increased migration. To identify the additional genes and pathways required to initiate tumorigenesis, we performed RNAseq to compare the transcriptomes of each cell line. Differentially expressed (DE) genes were identified using statistical methods based on generalized linear models to test for significant changes in gene expression (DKI and single mutant versus wild type, and DKI versus wild type or single mutants; FDR < 0.05) using EdgeR [18]. A total of 10,553 transcripts were found to be differentially expressed between the three cell lines, with the largest differences observed between wild type and knock-in of *KRAS* (G12V) alone, or wild type and knock-in of both mutations (Figure 2A). A heat map showing the top 50 upregulated genes in mutant cells highlights the marked differences in gene expression induced by a single base change in *KRAS* or *PIK3CA* (Figure 2B). Highlighted genes fall into the top 20 DE genes for the comparison of DKI versus wild type, or DKI versus each single mutant (Table 1). The majority of these top-ranked genes fall into the group DKI versus KRAS (G12V) (Table 1). RNAseq data were validated by RT-qPCR using primers specific to a selection of top upregulated genes (Figure 2C), and the majority of these genes showed significant upregulation in DKI cells under these test conditions.

These changes were brought about solely by the knock-in of each oncogene, since whole genome sequencing (WGS) (described in SI Materials and Methods) revealed no other mutations in DKI cells that have also been found to be mutated in a significant fraction of human tumors (COSMIC v70; cancer.sanger.ac.uk/cancergenome/projects/cosmic/) [19]. Copy number changes between wild type and mutant cells were determined as per Hart, J.R. et al. [20]. As described for PIK3CA (H1047R) cells previously, an analysis of amplifications and deletions using SNP heterozygosity revealed alterations in the variant allele frequency in chromosomes 5, 16, 19, 22, and X in mutant versus parental MCF-10A cells (Figure S5) [20]. DKI and PIK3CA (H1047R) cells showed an alteration at the 5′ end of chromosome 5 consistent with amplification of 5p13-15 (Figure S5A). In contrast, KRAS (G12V) and DKI cells contain multiple amplifications in chromosome 16 (Figure S5B) and chromosome 22 (Figure S5D). The amplifications at the 5′ end of chromosome 22 are reminiscent of wild type MCF-10As, while PIK3CA (H1047R) cells have two copies (Figure S5D). Additionally, WGS revealed amplifications in the 3′ end of chromosome 19 in KRAS (G12V) cells (Figure S5C), and the X chromosome in PIK3CA (H1047R) cells shows a loss of heterozygosity consistent with loss of a complete copy of the chromosome (Figure S5E). Overall, WGS showed that while the MCF-10A cells are not strictly isogenic, these alterations in single mutant cells cannot induce tumorigenesis.

To understand the functional significance of these changes, we performed Gene Set Enrichment Analysis (GSEA) using the highest probability-ranked transcripts to compute overlaps with Hallmark gene signatures [21,22]. GSEA revealed enrichment in DKI cells for genes associated with cell cycle-related targets of E2F transcription factors, progression through the G2/M cell cycle checkpoint and KRAS activation in comparison to wild type or to each single mutant cell line (Table 2). Both DKI and KRAS (G12V) cells showed enrichment for epithelial-mesenchymal-transition (EMT) and inflammatory response genes when compared to wild type cells, while DKI and PIK3CA (H1047R) cells showed enrichment for genes involved in hedgehog signaling (Table S1). Notably, DKI cells were enriched over both PIK3CA (H1047R) and KRAS (G12V) cells for MYC target genes (Figure 2D). Overall, RNAseq and GSEA analyses suggested that oncogenic PIK3CA and KRAS cooperate to transform cells via the upregulation of genes involved in progression through the cell cycle, EMT as well as through transcriptional targets of MYC.

### 2.3. Oncogenic PIK3CA and KRAS Induce the Expression of the Bromodomain-Containing Protein BRD9

**Figure 3:**
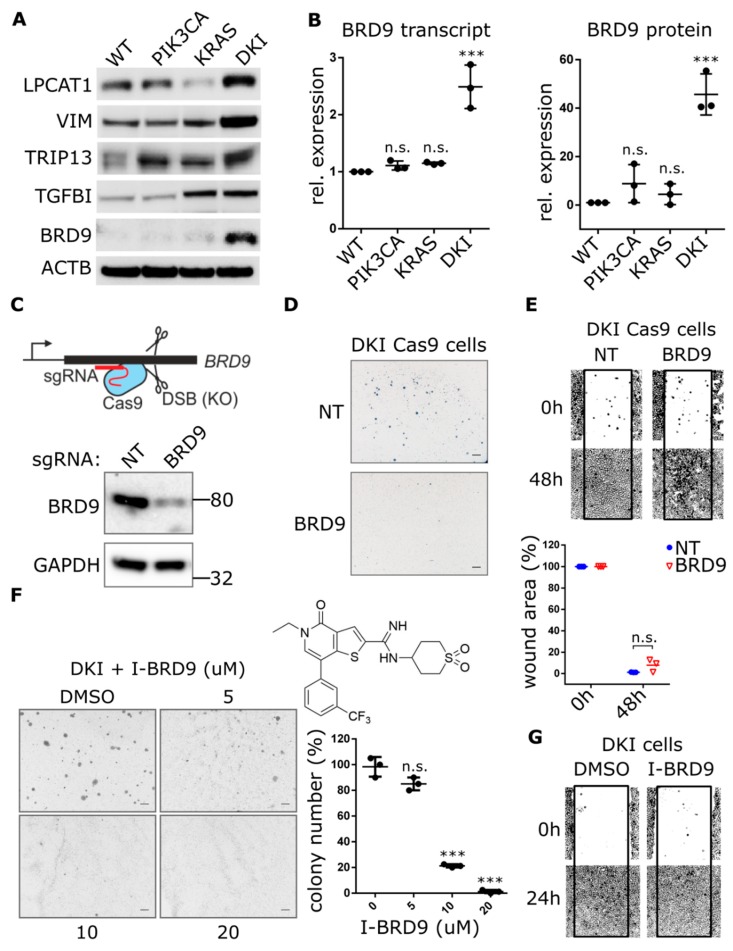
Genetic and pharmacological inhibition of BRD9 hampers the anchorage-independent growth but not migration of DKI cells. (**A**) Western blot analysis comparing the expression of indicated proteins in MCF10A WT and mutant cells. (**B**) Quantification of BRD9-mRNA by RT-qPCR (Mean ± SD, left panel) and BRD9 protein levels by immunoblotting (Mean ± SD, right panel) in MCF-10A WT and mutant cells. (**C**) Cartoon depicting CRISPR-Cas9-mediated gene knockout through DNA double-strand breaks and formation of insertions/deletions which disrupt the open reading frame. Lower panel: Immunoblot analysis of DKI cells transduced with CRISPR-Cas9 and sgRNA targeting BRD9. (**D**,**E**) Soft agar colony formation after 21 days and migration across a scratched wound over 48 h, by DKI-Cas9 cells expressing BRD9 or NT sgRNA. Lower panel: Quantitation of wound area (Mean ± SD). (**F**) Soft agar colony formation after 15 days by DKI cells in 0.3% agar supplemented with I-BRD9. Right panel: Colony number vs. dose I-BRD9 (Mean ± SD). (**G**) Migration across a scratched wound over 48 h by DKI cells treated with 20 µM I-BRD9. ***, *p* < 0.001 (unpaired *t*-test); n.s., not significant; NT, non-targeting sgRNA; scale bars = 100 μm.

A comparison of top DE genes between DKI and single mutant versus wild type showed that DKI and KRAS (G12V) cells share a large proportion of upregulated transcripts (Table S2). These genes fall largely within Hallmark gene sets encoding targets of E2F and genes involved in EMT. In order to identify the additional genes that KRAS (G12V) cells require to become oncogenic, we focused on the top DE genes upregulated in the DKI versus KRAS (G12V) group (Table 1). Immunoblots revealed only three corresponding proteins consistently upregulated in DKI over KRAS (G12V) cells: Lysophosphatidyl choline acyltransferase 1 (LPCAT1), Vimentin (VIM), and BRD9 (Figure 3A). These three proteins all fall within the top ten DE genes for the comparison of DKI versus KRAS (G12V) (Table 1) and are also encoded within the chromosome region 5p that showed copy number gains in PIK3CA (H1047R) and DKI cells by WGS (Figure S5A).

Since GSEA revealed DKI cells were uniquely enriched for MYC target genes, we turned our attention to BRD9. This protein is part of the human bromodomain family of epigenetic reader modules that selectively recognize acetylated lysines on histone tails [23]. It has recently been identified as a component of the SWI/SNF chromatin-remodeling complex, bound to the BRG1 ATPase subunit, and able to support the proliferation of myeloid leukemia cells via sustained *MYC* transcription [24]. We observed that BRD9 was significantly upregulated at the transcript and protein levels in DKI cells (Figure 3B). CRISPRi-mediated knockdown of *PIK3CA* or *KRAS* in DKI cells reduced *BRD9* transcript levels (Figure S6A), but BRD9 protein levels were more sensitive to reduction of *PIK3CA* in DKI (Figure S6B) as well as single mutant cells (Figure S6C). These observations led us to hypothesize that BRD9 may be acting to enhance *MYC* transcription in DKI cells, with MYC target genes then acting in concert with *KRAS* (G12V)-induced upregulation of E2F and EMT genes, to ultimately transform MCF-10A cells.

To test this hypothesis, we used lentiviral CRISPR-Cas9 constructs to knockout *BRD9* in DKI cells and assessed their ability to form colonies in soft agar. *BRD9* exon-specific sgRNAs that caused significant decreases in BRD9 protein levels (Figure 3C and Figure S7A) also abrogated soft agar colony formation (Figure 3D). This was also observed in MCF7 cells, where CRISPR-Cas9-mediated knockout of BRD9 (Figure S7B) resulted in loss of anchorage-independent growth (Figure S7C). In contrast, DKI-Cas9 cells expressing this same sgRNA showed no difference in migration when compared to cells expressing a NT guide (Figure 3E). These data were supported by use of the BRD9 inhibitor (I-BRD9) that shows >1000-fold higher selectivity for BRD9 over BRD4 bromodomains [24]. Dose-response effects on MCF-10A proliferation showed wild type, PIK3CA (H1047R) and DKI cells were equally inhibited, while KRAS (G12V) cells were most sensitive to I-BRD9 (Figure S8A). Still, soft agar growth was abolished at doses 35-fold less than the observed IC50 for DKI cell proliferation (Figure 3F). Consistent with CRISPR-Cas9-mediated knockout of BRD9, pharmacological inhibition of BRD9 did not change migration capacity in DKI cells (Figure 3G) as well as in wild type, PIK3CA (H1047R) and KRAS (G12V) cells treated with IC50 concentrations of I-BRD9 for 24 h (Figure S8B).

**Figure 4:**
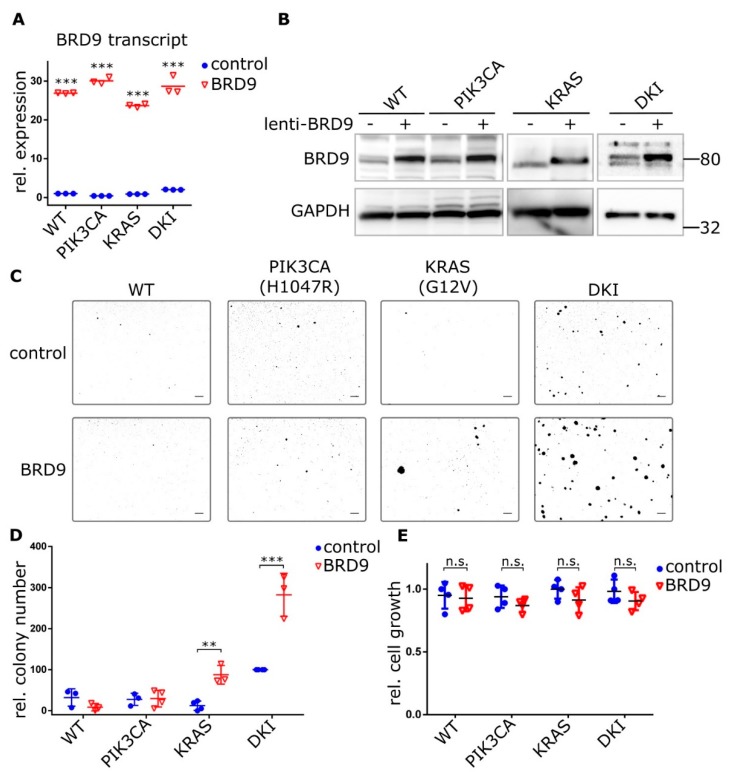
Ectopic BRD9 expression induces anchorage-independent growth in KRAS (G12V) single mutant cells and strongly increases it in DKI cells. (**A**) RT-qPCR (Mean ± SD) and (**B**) immunoblot data showing BRD9 overexpression from a lentiviral construct in MCF-10A cells, empty vector was used as control. (**C**) Soft agar colony formation after 21 days by MCF-10A WT and mutant cells under puromycin selection for stable expression of BRD9. (**D**) Quantification of soft agar growth (Mean ± SD). (**E**) Proliferation of MCF-10A WT and mutant cells expressing control (empty) or BRD9 lentivirus. Alamar Blue assay 72 h after seeding (Mean ± SD). **, *p* < 0.01; ***, *p* < 0.001 (unpaired *t*-test); n.s., not significant; scale bars = 100 μm.

To test whether exogenous BRD9 expression could induce an anchorage-independent growth phenotype in single mutant cells, we overexpressed BRD9 in wild type and mutant MCF-10A cells using lentiviral constructs (Figure 4A,B). PIK3CA (H1047R) and KRAS (G12V) cells stably expressing BRD9 formed discreet colonies after 21 days growth in 0.3% agar, while no colonies were observed with wild type cells over-expressing BRD9 or empty vector control cells (Figure 4C). This growth reached significance versus empty vector control with KRAS (G12V) and DKI cells only (Figure 4D). Importantly, average colony size was also increased in KRAS (G12V) cells (Figure 4C). Despite the stark contrast in soft agar colony growth, overexpression of BRD9 did not increase cell proliferation (Figure 4E). Overall, we observed that BRD9 is essential for the anchorage-independent growth phenotype that defines oncogenic *PIK3CA* and *KRAS* cooperativity, and that supplementing BRD9 in KRAS (G12V) cells partially rescues the necessity for anchorage-dependent growth by these cells.

### 2.4. BRD9 is Part of the SWI-SNF Complex and Augments MYC Activity

**Figure 5:**
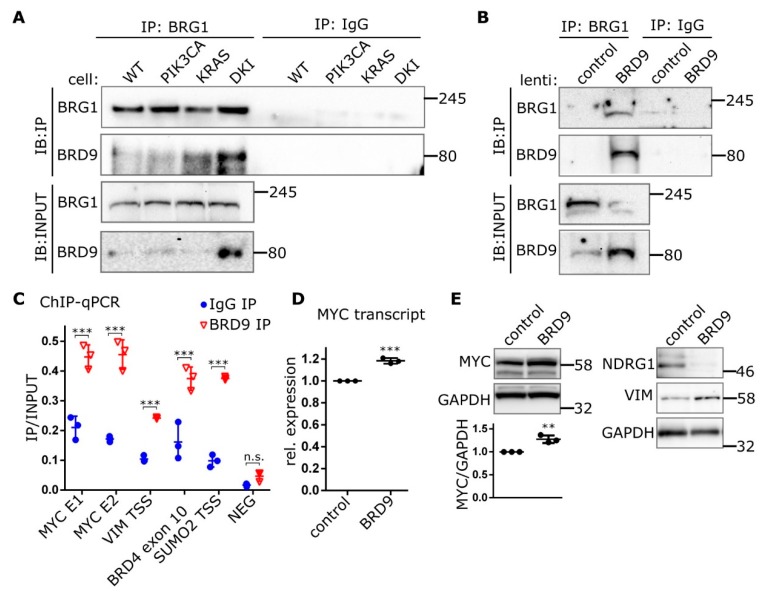
BRD9 is part of the SWI-SNF complex and binds MYC enhancer sequences. (**A**) Immunoblots showing Co-IP of BRD9 with the SWI-SNF subunit BRG1 from MCF-10A nuclear extracts or (**B**) nuclear extracts isolated from DKI cells transduced with lenti-BRD9. (**C**) ChIP data (Mean ± SD) showing BRD9 occupancy at MYC super-enhancer and VIM promoter DNA regions. BRD4 and SUMO2 represent positive control sequences; NEG represents a non-specific negative control sequence. (**D**) RT-qPCR (Mean ± SD) of MYC transcript in DKI cells transduced with empty (control), or BRD9-encoding lentiviral constructs. (**E**) Immunoblot data showing MYC (with densitometric quantification, lower panel), target NDRG1, and VIM expression in DKI cells transduced with empty (control), or BRD9-encoding lentiviral constructs. **, *p* < 0.01; ***, *p* < 0.001 (unpaired *t*-test); n.s., not significant.

The multi-subunit SWI-SNF chromatin-remodeling complex has been implicated in various human cancers. The composition of this complex varies depending on tissue and cell type, with BRD7, BRD9, BRM and PBRM1 all having been shown to be potential chromatin reader domains in the SWI-SNF complex. The ATPase subunit BRG1 has been shown to bind BRD9 in acute myeloid leukemia (AML) cells and is essential in supporting enhancer-mediated *MYC* expression in this context [24]. Here, we show that endogenous BRG1 co-immunoprecipitates BRD9 from MCF-10A nuclear lysates, with the most BRD9 being recovered from DKI nuclear lysates (Figure 5A). Congruently, BRG1 appears in pull-downs of endogenous BRD9 (Figure S9). Immunoprecipitations of BRG1 from nuclear lysates of DKI cells overexpressing BRD9 also recovered large amounts of BRD9 compared to control cells (Figure 5B). ChIP experiments showed that BRD9 was enriched at sequences found within the *MYC* super-enhancer region [24], 1.7 Mb downstream of the *MYC* promoter (Figure 5C). BRD9 was also found at the *VIM* promoter as well as at *BRD4* and *SUMO2* (Figure 5C), reported BRD9-interacting genes based on data from curated pathway/protein-interaction databases and text-mining [25]. MYC protein levels were also increased in DKI cells, and the MYC repression target NDRG1 was decreased (Figure S10A). In DKI cells overexpressing BRD9, *MYC* mRNA (Figure 5D) and protein levels are increased and the NDRG1 protein is similarly downregulated (Figure 5E). These data show that BRD9 is part of the SWI-SNF chromatin remodeling complex in MCF-10A cells and that it can be found on *MYC* enhancer sequences and at the *VIM* promoter. In these same cells, VIM and MYC are upregulated, suggesting a link between these proteins and BRD9 levels.

### 2.5. BRD9 Inhibition Blocks MYC Expression and MYC Inhibition Blocks Anchorage-Independent Growth

**Figure 6:**
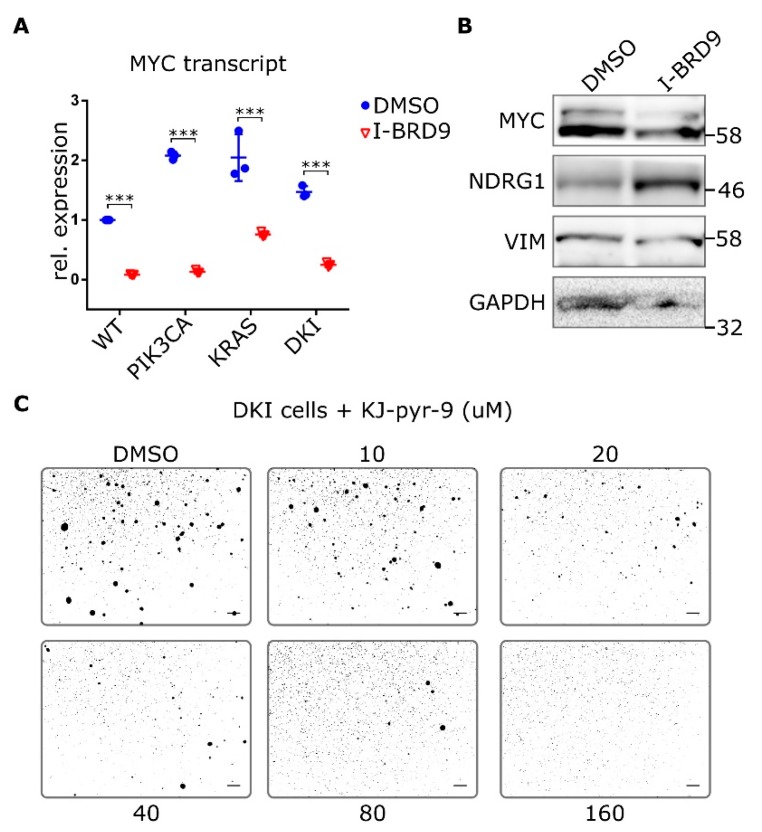
Inhibition of MYC blocks anchorage-independent growth in double mutant cells. (**A**) RT-qPCR data (Mean ± SD) showing MYC expression in MCF-10A WT and mutant cells after 24 h incubation with 20 µM I-BRD9. (**B**) Protein levels of MYC and target NDRG1 as well as VIM in DKI cells after 24 h treatment with 20 µM I-BRD9. (**C**) Soft agar growth of DKI cells in the presence of the MYC inhibitor KJ-Pyr-9 for 15 days. ***, *p* < 0.001 (unpaired *t*-test); scale bars = 100 μm.

To better understand whether BRD9 affects MYC expression, we looked at MYC transcript and protein levels after treatment with I-BRD9. Transcript levels of *MYC* were reduced in all MCF-10A cells after treatment with I-BRD9 for 24 h (Figure 6A). Likewise, protein levels of MYC decreased in DKI cells after I-BRD9 treatment and NDRG1 was upregulated (Figure 6B). VIM levels in DKI cells were also reduced after I-BRD9 treatment (Figure 6B and Figure S10B). Finally, inhibition of MYC with the small molecule KJ-Pyr-9 [26] was also able to diminish DKI soft agar colony formation (Figure 6C). Thus, expression of MYC and its target genes is related to BRD9 function, and MYC function is necessary to maintain anchorage-independent growth.

## 3. Discussion

PI3K pathway activation is one of the most common events in human cancer, functioning to promote cell growth and proliferation as well as survival through several downstream targets. Similarly, KRAS is often mutated in human cancers, and it drives similar growth and proliferation phenotypes. Despite acquisition of these growth-promoting phenotypes, cells require more than one driver mutation to produce a molecular profile capable of sustaining tumor growth. The MCF-10A system is an instructive model to study the complementing contributions of two oncogenes. Individually, *KRAS* and *PIK3CA* mutations impart features of transformation, such as EGF-independent growth, yet this is not sufficient for tumorigenicity. This is true even in the context of background mutations in MCF-10A wild type cells, which include deletion of the tumor suppressor *CDKN2A* and amplification of MYC [27,28,29]. However, only their combination results in true oncogenic transformation capable of producing colony formation in soft agar as well as xenograft tumors in mice. Using transcriptomic profiling, we identified downstream targets of oncogenic *PIK3CA* and *KRAS* that are required to induce tumorigenesis. We found that double mutant cells were enriched for genes that characterize a MYC target signature when compared to either single mutant cell line. This gene signature was mediated in part by BRD9 that was found in the SWI-SNF chromatin-remodeling complex in MCF-10A cells, bound to the *MYC* super-enhancer locus. Furthermore, BRD9 was essential for the anchorage-independent growth phenotype that distinguishes double mutant from wild type and single mutant MCF-10A cells.

The mechanisms allowing a single oncogene to elicit a transformed phenotype have been demonstrated in various ways. Tumors that arise in mice with a single mutant allele of *KRAS* were shown to have increased copy numbers of the mutant allele [30]. In contrast, knock-in of single copies of either mutant *KRAS* or *PIK3CA* showed no obvious phenotype in human breast epithelial cells or mouse liver [16,31,32]. Single copies of mutant *KRAS* or *PIK3CA* thus require a second oncogene to fully transform cells. Multiple-lentiviral-expression systems have been used to generate sarcoma mice models by combining *HRAS* (G12V) with knockdown of p53 or CDKN2a [33]. Additionally, it has long been known that MYC cooperates with RAS to transform cells [34,35]. Likewise, *PIK3CA* (H1047R)-driven mouse mammary tumors often recur in the presence of *c-myc* amplification, and this combination is observed in a substantial fraction of human breast tumors [36,37].

In this study, soft agar growth was observed only with MCF-10A cells expressing both oncogenic *KRAS* and *PIK3CA*. This is consistent with original studies using these cells, which also showed that disrupting the Ras-binding domain of p110α caused attenuation but not abrogation of the proliferative effects of oncogenic PIK3CA and KRAS [16,38]. This suggests that direct KRAS/p110α interaction is only partially responsible for the tumorigenic phenotype observed with DKI cells. We showed that stable knockdown of *PIK3CA* or *KRAS* had distinct effects: Growth in soft agar depended largely on *PIK3CA*, while migration depended on *KRAS*. This is in line with our GSEA data that showed that both *KRAS* (G12V) and DKI cells are enriched for EMT and cell cycle-related genes, while *PIK3CA* (H1047R) and DKI cells are enriched for hedgehog and PI3K-AKT-MTOR signaling genes. This implies that cell motility and cytoskeletal reorganization only partly contribute to anchorage-independent growth.

DKI, *KRAS* (G12V) and *PIK3CA* (H1047R) cells show MAPK pathway activation as evidenced by an increase in phosphorylated ERK1/2. It is unclear how MAPK activation is achieved in *PIK3CA* (H1047R) cells, but we note that the *GRB2*, *RSKα1*, and *RAF1* genes all fall within the leading-edge subset of PI3K-AKT-MTOR signaling enriched in these cells. These genes link EGF receptor activation to MAPK signaling via RAS and may account for the enhanced MAPK activity seen in *PIK3CA* (H1047R) cells. Whole genome sequencing also revealed that *PIK3CA* (H1047R) and DKI cells show copy number gains in chromosome region 5p relative to the parental MCF-10A cell line. This region encodes the genes for *BRD9, TRIP13, LPCAT1, CLPTM1L*, and *NSUN2*, all genes that are upregulated in DKI versus *KRAS* (G12V) cells as shown by RNAseq and RT-qPCR. These genes have been linked to various cancers, yet their presence in *PIK3CA* (H1047R) cells is not sufficient to induce anchorage-independent growth. In DKI cells, BRD9 protein expression is upregulated over TRIP13, LPCAT1, and NSUN2 and is significantly higher than seen in either single mutant cell line. The mechanism underlying these differences in BRD9 protein levels would be interesting to explore further but suggest a distinctive demand for BRD9 in DKI cells. Crucially, we observed that stable knockdown of *BRD9* in DKI cells by CRISPR-Cas9 blocked soft agar colony formation but not migration. This paralleled our results seen with knockdown of *PIK3CA* suggesting that oncogenic p110α signaling is an important contributor to anchorage-independent growth. Still, complementary rescue of single mutant cells with ectopic expression of BRD9 showed only partial restoration of soft agar growth by *KRAS* (G12V) cells but not of *PIK3CA* (H1047R) cells. This indicates that while BRD9 is necessary for anchorage-independent growth, additional contributions from the *KRAS* (G12V) gene signature are vital for transformation. These findings suggest an investigation of a possible role of BRD9 in human cancers that carry both a KRAS and PI3K mutation.

BRD9 was recently identified as a component of the SWI/SNF chromatin-remodeling complex, bound to the ATPase subunit BRG1 [24]. Hohmann et al. showed that AML cells were uniquely dependent on BRD9 to support the proliferation via sustained *MYC* transcription [24]. Loss-of-function studies originally defined a tumor suppressor role for the SWI/SNF complex in human cancers [39,40], but more recently the complex has been shown to be critical for directing the oncogenic transcriptional program of leukemia cells [24,41]. Bromodomains are the only chromatin interaction modules that specifically recognize and bind to ε-N-acetylated lysines of histones [23,42]. MYC transcription is associated with increases in histone lysine side-chain acetylation, and inhibition of bromodomain and extra-terminal domain (BET) proteins can repress MYC expression and MYC-dependent target gene expression [43,44,45]. BRD4 has most often been associated with MYC expression, with this regulation seen in multiple myeloma, AML and ER+ breast cancer [44,46,47]. Both BRD9 and BRD4 are enriched at *MYC* enhancers in AML cells, despite being phylogenetically distinct and having differing binding preferences for acetylated lysines in vitro [41,42]. We did not test for BRD4 occupancy at *MYC* in MCF-10A cells but note that *BRD4* was not enriched in DKI cells over wild type or single mutant cells.

To understand the growth effects of BRD9 in MCF-10A cells, we asked whether BRG1 associates with BRD9 and the *MYC* enhancer loci. Immunoprecipitation of endogenous BRG1 showed that BRD9 is part of the SWI-SNF complex in all MCF-10A varieties used in this study. Significantly, we also found that BRD9 enriches for the *MYC* “super-enhancer” locus originally described by Shi et al. and not the proximal enhancer sites [41]. This super-enhancer is a cluster of binding elements that loop over a 1.7 Mb distance to contact the *MYC* promoter, and both BRG1 and BRD9 were found to be essential for maintaining transcription factor occupancy and *MYC* promoter communication in leukemia cells [24,41]. Pharmacological inhibition of BRD9 reduced *MYC* transcript and protein levels and increased levels of NDRG1. Inhibiting either BRD9 or MYC also decreased the capacity of DKI cells to grow in soft agar. We cannot exclude the possibility that BRD9 functions independently of SWI-SNF, particularly because BRD9 was found bound to BRG1 in *KRAS* (G12V) as well as DKI cells. In ChIP experiments, we observed BRD9 binding at the TSS of the *VIM* gene. We also see high levels of VIM in DKI cells and cells overexpressing BRD9. Interestingly, loss of BRG1 induced metastasis in human lung cancer cells by driving E-cadherin loss and VIM upregulation [48]. BRD9 may therefore provide a link between the enrichment of EMT-related genes and MYC target genes observed in DKI cells by RNAseq and GSEA. While MYC is rarely mutated in cancer, most tumors are dependent on MYC for cell division and survival, and MYC amplification is necessary for tumor formation in nude mice [36,49]. Since MYC amplification has been reported in MCF-10A cells, the additional essential factor driving tumorigenesis in this context may be an addiction to SWI/SNF activity via increased levels of BRD9 at *MYC* enhancers.

## 4. Materials and Methods

### 4.1. Cell Culture and Viability Assays

MCF-10A wild type and derivative knock-in cells were a generous gift from Dr. Ben Ho Park (Johns Hopkins University, Baltimore, MD, USA). MCF-10A cells were cultured in DMEM/F12 (Gibco, Thermo Fisher Scientific, Asheville, NC, USA) supplemented with 5% horse serum (Gemini, New York, NY, USA), 10 µg/mL insulin (Sigma, St. Louis, MO, USA), 0.5 µg/mL hydrocortisone (Sigma), 0.1 µg/mL cholera toxin (Sigma) and 1% penicillin/streptomycin/L-glutamine (Sigma). Wild type cells were supplemented with an additional 20 ng/mL EGF (Repligen, Waltham, MA, USA). Assay conditions were in the absence of EGF, unless otherwise noted. MCF7 cells (ATCC HTB-22) were cultured in DMEM (Gibco) supplemented with 10% FBS and 0.01 mg/mL insulin. HEK293T cells (ATCC CRL-3216) were cultured in DMEM containing L-glutamine (Gibco) supplemented with 10% FBS. All cells were maintained at 37 °C with 5% CO2, passaged at subconfluence using 0.25% trypsin EDTA (Life Technologies, Carlsbad, CA, USA) and discarded after passage 15. Cells stably expressing Cas9 or dCas9/KRAB along with sgRNA were selected and maintained in 3 ug/mL puromycin (Sigma).

Inhibition of p110α, MEK and BRD9 was achieved using A66 (Tocris, Minneapolis, MN, USA), AZD6244 (Selleck Chemicals, Houston, TX, USA) and I-BRD9 (Tocris), respectively. MYC was inhibited using KJ-Pyr-9, kindly supplied by Dr. Kim Janda (The Scripps Research Institute, La Jolla, CA, USA). For cell viability assays, exponentially growing cells were seeded into 96-well plates in complete media at a density of 1500 cells/well. The media was exchanged 24 h later for EGF-free media +/- inhibitor. After 72 h growth, cells were washed with PBS and stained with 10 µg/mL Resazurin (Sigma). Cell viability was assessed by quantifying the fluorescent, reduced intermediate of the dye at 595 nm on a plate reader. For growth on a non-binding surface, 96-well plates were coated with 20 mg/mL poly2-hydroxymethacrylate (polyHEMA) (Sigma) in 95% ethanol. Plates were dried overnight in a non-humidified 37 °C incubator prior to UV sterilization for 30 min. Exponentially growing cells were resuspended at 5 × 10^4^ cells/mL complete EGF-free growth media, and 200 µL were plated per well to yield 10,000 cells per well. Cell viability was quantified after 72 h with Resazurin dye as above, or inhibitor was added and incubated for an additional 72 h at 37 °C prior to addition of Resazurin.

### 4.2. Next-Generation Sequencing

MCF-10A cells were plated in 6 cm tissue culture dishes in triplicate using complete DMEM/F12 and cultured overnight. The media was then exchanged for fresh MCBD-170 media [20] and cultured for 24 h. Cells were harvested with trypsin followed by soybean trypsin inhibitor (Thermo Fisher Scientific, Asheville, NC, USA) and RNA isolated using the Maxwell RSC simplyRNA cells kit (Promega, Madison, WI, USA) as per the manufacturer’s instructions. DNA was isolated in parallel using Ultra-Pure phenol:chloroform:isoamyl alcohol (Thermo Fisher Scientific). Nucleic acid purity and integrity was confirmed by agarose gel and sent to Genewiz (South Plainfield, NJ, USA) for RNAseq and WGS. Raw and processed data are available online (Gene Expression Omnibus accession no. GSE128388).

### 4.3. Gene Expression Analysis

RNAseq data were mapped to HG38 using STAR aligner [50]. Reads were counted using htseq [51] utilizing the GENCODE version 27 gene annotations [52,53]. Analysis of differential expression was performed using edgeR [18] after filtering the data such that a minimum of three samples had more than 0.3 reads per million. GSEA was performed using GSEA2 [21] with the MsigDB Hallmark gene sets (*R* = 10,000) [22].

### 4.4. Plasmids and CRISPR-Cas9 Gene Editing

For stable SpCas9 and sgRNA expression in MCF-10A cells, we used lentiCas9-Blast and lentiGuide-Puro (provided by Feng Zhang, Addgene plasmids #52962 and #52963, respectively). The vector for expression of catalytically inactive SpCas9 (dCas9) was generated by introducing the mutations D10A and H840A by site-directed mutagenesis using lentiCas9-Blast as a template. Lenti-dCas9-KRAB-Blast was prepared by ligating the coding sequence of amino acids 1 to 72 of human ZNF10 into the unique *BamH*I site of lenti-dCas9. *KRAS* and *PIK3CA* were knocked-down by lentiviral transduction of gene-targeting sgRNAs into MCF-10A cells stably expressing lenti-dCas9-KRAB-Blast. *BRD9* was knocked-out by lentiviral transduction of gene-targeting sgRNAs into MCF-10A cells stably expressing lentiCas9-Blast. To obtain lenti-BRD9-Puro for overexpression studies, the *BRD9* coding sequence was PCR-amplified from MCF-10A DKI cell cDNA and cloned into a lentiviral backbone in frame with a P2A-autoprotease sequence and a puromycin resistance gene. Lentiviral particles were produced by polyethylenimine (PEI)-mediated transfection of HEK293T cells with the specific transfer vector along with Gag/Pol, Rev and VSVG packaging vectors. Monoclonal cell populations were isolated by limiting dilution and stable cell lines generated under puromycin or blasticidin selection prior to analysis of expression by immunoblotting. Single guide RNAs were ordered as oligonucleotides from Integrated DNA Technologies (Coralville, IA, USA) and are listed in Table S3.

### 4.5. RT-qPCR

Total RNA was extracted using the Maxwell RSC simplyRNA cells kit (Promega), and cDNA was synthesized using the High Capacity cDNA Reverse Transcription Kit, according to the manufacturer’s instructions (Thermo Fisher Scientific). All results were quantified by qPCR using Power SYBR green (Thermo Fisher Scientific) on a Roche LightCycler 96 Instrument. Fold change in gene expression was calculated using the Livak (2^−ΔΔCt^) method with RPLP0 as the reference gene [54]. Primers are listed in Table S4.

### 4.6. Immunoblotting

Cells were seeded at equal densities in complete media and the media exchanged 24 h later for EGF-free media +/− inhibitor for an additional 24 h growth. Cells were scraped into 1X PBS and lysed in RIPA buffer containing 1 mM PMSF (Sigma), 1 mM Na_3_VO_4_ (Sigma) and 1x cOmplete Protease Inhibitor cocktail (PI) (Roche, Indianapolis, IN, USA). Twenty milligrams protein was resolved by SDS-PAGE using NuPAGE 4–12% Bis-Tris protein gels (Life Technologies), transferred onto PVDF (Millipore, Billerica, MA, USA) and blocked in 5% BSA. Following overnight blotting with primary antibody, protein was detected by ECL using horseradish peroxidase-conjugated secondary antibodies (Sigma). Primary antibodies used in this study are: anti-RAS (#3965), anti-phospho-p44/42 MAPK (ERK1/2) (Thr202/Tyr204) (#4370S), anti-p44/42 MAPK (ERK1/2) (#4696), anti-PI3K p110α (#4255), anti-phospho-AKT (Ser473) (#4051), anti-AKT (#4685), anti-C-MYC (#9402), anti-NDRG1 (#5196), anti-GAPDH (#2118) (Cell Signaling Technologies, Danvers, MA, USA), anti-BRD9 (#A303-781A, Bethyl Laboratories, Montgomery, TX, USA), anti-BRG1 (#sc-17796) and anti-VIM (#sc-6260) (Santa Cruz Biotechnology, Santa Cruz, CA, USA).

### 4.7. Immunoprecipitation

To isolate nuclear extracts, cell pellets were washed with ice cold 1X PBS, resuspended in 500 µL hypotonic buffer (10 mM HEPES-KOH pH 7.9, 1.5 mM MgCl_2_, 10 mM KCL, 1 mM DTT and 1X PI) and incubated on ice for 30 min. Nuclei were separated by addition of 20 µL 0.05% NP-40 and centrifugation at 720× *g* for 5 min at 4 °C. Nuclei were resuspended in 100 µL nuclear extraction buffer (20 mM Hepes-KOH pH 7.9, 25 % glycerol, 420 mM NaCl, 1.5 mM MgCl_2_, 0.2 mM EDTA, 1 mM DTT and 1X PI) and incubated on ice for 30 min, vortexing every 5 min. Debris was pelleted at 16,000× *g*, 4 °C for 10 min. The resulting supernatant was diluted to 150 nM NaCl with nuclear extraction buffer without NaCl. To immunoprecipitate the SWI/SNF complex, 1 ug BRG1 antibody (Santa Cruz #sc-17796) or control IgG antibody (Thermo Fisher Scientific) were incubated with ~1 mg nuclear extract overnight at 4 °C. The antibody-antigen complex was pulled down using IgG pre-blotted Protein G Sepharose 4 Fast Flow resin (GE Healthcare, Marlborough, MA, USA) for 2 h at 4 °C. To remove non-specific binders, the bead-immune complex was washed three times with IP wash buffer (Tris buffer pH 7.4, 300 mM NaCl, 0.5 % NP-40, 1 mM DTT and 1X PI) and once with 1X PBS. Immune complexes were eluted with Bolt LDS sample buffer containing Bolt sample reducing agent (Life Technologies), boiling samples for 5 min, prior to immunoblotting for BRG1 and BRD9.

### 4.8. Chromatin Immunoprecipitation

Subconfluent cells (2 × 10^7^ cells per sample) were washed with 1X PBS and crosslinked with 1% formaldehyde at room temperature for 20 min. Crosslinking was quenched by adding 1.5 M glycine to a final concentration of 0.2 M and rocking gently for 10 min at room temperature. Cells were then rinsed twice with ice cold 1X PBS, harvested with a cell scraper and pelleted at 240× *g* for 8 min at 4 °C. Nuclei were isolated by incubating cells in 1 mL hypotonic cell lysis buffer (10 mM TRis pH 8.0, 10 mM NaCl, 0.2% NP-40, 1X PI) on ice for 15 min and pelleting nuclei at 600× *g* for 5 min at 4 °C. Nuclei were lysed with 200 µL nuclear lysis buffer (50 mM Tris pH 8.0, 10 mM EDTA, 1% SDS, 1X PI) on ice for 30 min, vortexing every 5 min. Prior to sonication, nuclei were passed through a 25 G needle five times, avoiding foaming. Chromatin was reduced to <500 bp fragments by sonicating in an ice bath using a QSonica 700 instrument (amplitude 70%, 15s ON/45s OFF, 20 min total ON time). Debris was pelleted at 8000× *g* for 1 min at room temperature. 25 µg DNA was used per IP and diluted 1:10 with IP dilution buffer (20 mM Tris pH 8.0, 150 mM NaCl, 2 mM EDTA, 0.01% SDS, 1% Triton X-100, 1X PI). Diluted chromatin was precleared with Protein G Sepharose 4 Fast Flow resin (GE Healthcare) in IP dilution buffer for 1 h at 4 °C, prior to overnight incubation at 4 °C with 2 µg anti-BRD9 (Bethyl Laboratories #A303-781A) or control IgG antibody (Thermo Fisher Scientific). Immune complexes were collected with protein G beads for 2 h at 4 °C, washed twice with IP wash buffer 1 (20 mM Tris pH 8.0, 150 mM NaCl, 2 mM EDTA, 0.1% SDS, 1% Triton X-100, 1X PI), once with IP wash buffer 2 (20 mM Tris pH 8.0, 250 mM NaCl, 2 mM EDTA, 0.1% SDS, 1% Triton X-100), once with IP wash buffer 3 (10 mM Tris pH 8.0, 0.25 M LiCl, 1 mM EDTA, 1% NP-40, 1% deoxycholate) and twice with TE pH 8.0. To elute DNA, bead-immune complexes were resuspended in 150 µL IP elution buffer (0.1 M NaHCO, 1% SDS), incubated for 10 min at room temperature and pelleted at 2000× *g*. This was repeated and the supernatant combined. Sample volumes were adjusted to 500 µL and the crosslinks reversed by adding NaCl to 0.3 M and incubating at 65 °C overnight. DNA was isolated using Ultra-Pure phenol:chloroform:isoamyl alcohol (Thermo Fisher Scientific) and analyzed by qPCR. Relative enrichment was calculated by referencing each IP signal to an input standard curve dilution series (IP/Input). Primers are listed in Table S4.

### 4.9. Colony Formation in Semisolid Medium

For colony formation assays, 2 × 10^4^ exponentially growing cells were resuspended in 1 mL 0.3% SeaPlaque agarose (Sigma) in complete growth media, prepared as 2X from powdered DMEM/F12 (Gibco) and including EGF +/- inhibitor or selection antibiotic. The cell/agarose mix was then poured on top of a pre-warmed 2 mL base layer, comprising 0.6% agarose, in six-well tissue culture plates. The agarose was allowed to solidify at room temperature prior to incubation at 37 °C. A 1 mL feeder layer (0.3% agarose/growth media with EGF +/- inhibitor or selection antibiotic) was added to the wells twice a week. After 2–3 weeks incubation, the wells were photographed under phase contrast using a dissecting microscope and quantified using Image J software (NIH). At least two independent experiments were done in triplicate.

### 4.10. Statistical Analysis

P values were determined by One- or Two-way ANOVA using GraphPad Prism v.6 (San Diego, CA, USA).

## 5. Conclusions

In this study, we investigated the early events of cellular transformation mediated by mutant KRAS and PIK3CA in breast epithelial cells. Mutant PIK3CA induced proliferation and anchorage-independent growth, whereas activated KRAS appeared to primarily induce a more motile phenotype. Transcriptome analysis revealed that when compared to wild type or single knock-in, DKI cells were enriched for a MYC target gene signature. To understand how PIK3CA and KRAS promote MYC activity, we examined expression data and found BRD9 as a prominently up-regulated gene in DKI cells. CoIP experiments confirmed interaction between BRD9 and the SWI/SNF chromatin remodeling complex, and ChIP-qPCR experiments found BRD9 bound to the MYC super-enhancer locus.

Both, CRISPR-Cas9-mediated knock-out and pharmacological inhibition of BRD9 led to inhibition of MYC expression and blocked the oncogenic phenotype of DKI cells, whereas over-expression of BRD9 had the opposite effect. We conclude that BRD9 is a critical mediator of KRAS and PIK3CA oncogene cooperation to induce MYC activity. These results may have pharmacological significance to targeted treatment of tumors harboring these oncogenes.

## Figures and Tables

**Table 1:** Top 20 differentially expressed genes (upregulated), DKI vs. WT and single mutant cells. Log2FC sorted by *p* value.

Rank	DKI vs. WT	log2FC	DKI vs. PIK3CAH1047R	log2FC	DKI vs. KRASG12V	log2FC
1	COL4A1	4.19	DHRS3	5.40	CLPTM1L	1.08
2	TFPI2	4.09	TFPI2	4.29	CCT5	1.14
3	FAM110C	4.47	TGFBI	4.14	MRPS30	1.06
4	ARL4C	3.84	TINAGL1	4.04	NSUN2	1.01
5	TCF19	3.73	COL4A1	3.97	LPCAT1	1.06
6	DHRS3	5.33	FAM110C	3.78	BASP1	0.98
7	TINAGL1	4.18	TCF19	3.73	PEAR1	2.18
8	LMNB2	3.43	LMNB2	3.67	BRD9	1.07
9	PROSER2	3.42	MYBL2	3.59	VIM	1.22
10	ADAM19	4.66	CCT2	3.54	ANKH	1.16
11	PHLDA1	3.40	ADAM19	3.49	TRIP13	1.05
12	TGFBI	4.23	UBA2	3.44	IRX4	1.17
13	CCT2	2.80	SRP14	3.38	OTULIN	0.83
14	TXNRD1	2.91	EIF1	3.18	ICE1	0.94
15	UBA2	3.25	MCM2	3.17	TARS	0.93
16	TRIP13	3.44	RPL7	3.10	DNAJC21	1.03
17	LTBP1	3.06	NSUN2	3.09	LMBRD2	1.06
18	NSUN2	3.04	CSTA	2.99	ZNF622	0.93
19	MCM2	3.61	GLRX	2.93	CEP72	1.26
20	UHRF1	3.52	RPS7	2.86	KCNK6	1.03

**Table 2:** Selected Hallmark Gene Signatures significantly upregulated in MCF-10A DKI vs. WT and single mutant cells as determined by GSEA.

DKI vs. WT	DKI vs. PIK3CA_H1047R	DKI vs. KRAS_G12V
EPITHELIAL_MESENCHYMAL_TRANSITION	E2F_TARGETS	MYC_TARGETS_V1
E2F_TARGETS	G2M_CHECKPOINT	E2F_TARGETS
G2M_CHECKPOINT	EPITHELIAL_MESENCHYMAL_TRANSITION	OXIDATIVE_PHOSPHORYLATION
KRAS_SIGNALING_UP	KRAS_SIGNALING_UP	G2M_CHECKPOINT
APICAL_JUNCTION	MITOTIC_SPINDLE	MYC_TARGETS_V2
TNFA_SIGNALING_VIA_NFKB	ESTROGEN_RESPONSE	MTORC1_SIGNALING
MTORC1_SIGNALING	APICAL_JUNCTION	APOPTOSIS
TGF_BETA_SIGNALING	MTORC1_SIGNALING	UNFOLDED_PROTEIN_RESPONSE
WNT_BETA_CATENIN_SIGNALING	MYC_TARGETS_V1	
IL2_STAT5_SIGNALING	TNFA_SIGNALING_VIA_NFKB	

## Supplementary Materials

The following are available online at https://www.mdpi.com/2072-6694/11/11/1634/s1, Supplemental experimental procedures, Figure S1: DKI cells have activated PI3K and KRAS pathways and show synergistic growth effects, Figure S2: CRISPR-Cas9-mediated knockdown of *KRAS* and *PIK3CA* in MCF-10A DKI cells, Figure S3: Inhibition of KRAS or PI3K signaling limits anchorage-independent growth of MCF-10A DKI cells, Figure S4: Inhibition of MEK by AZD6244 does not impact cell viability, Figure S5: Large CNV determination in MCF-10A cells by SNP analysis after whole genome sequencing, Figure S6: BRD9 transcript is downregulated after CRISPR inactivation of *KRAS* and *PIK3CA*, Figure S7: CRISPR-Cas9 knockdown of BRD9 inhibits MYC and blocks anchorage-independent growth, Figure S8: Small molecule inhibition of BRD9 has no effect on migration capacity, Figure S9: BRG1 co-immunoprecipitates with endogenous BRD9, Figure S10: MYC and BRD9 are upregulated in MCF-10A DKI cells, Table S1: Top ranked Hallmark gene signatures for MCF-10A mutant vs. WT determined by GSEA, Table S2: Top 30 DE genes in mutant vs. WT cells, Table S3: Single guide RNA oligo sequences, Table S4: PCR primer sequences. References [55,56,57,58,59] are cited in the supplementary materials.
