# Supplementary material for: PIK3CA Cooperates with KRAS to Promote MYC Activity and Tumorigenesis via the Bromodomain Protein BRD9
Source: Cancers (Basel). 2019 Oct 24;11(11):1634. doi: 10.3390/cancers11111634 (PMC6896067; doi:10.3390/cancers11111634)
Supplement: Supplementary file 1 [file cancers-11-01634-s001.pdf]

Supplementary materials

PIK3CA cooperates with KRAS to promote MYC activity and tumorigenesis via the bromodomain protein BRD9

Catherine M. Bell, Philipp Raffeiner, Jonathan R. Hart, Peter K. Vogt

Department of Molecular Medicine, The Scripps Research Institute, 10550 North Torrey Pines Road, La Jolla, CA 92037, USA

Materials included:

- Supplementary experimental procedures

- Figure S1: DKI cells have activated PI3K and KRAS pathways and show synergistic growth effects.
- Figure S2: CRISPR/Cas9-mediated knockdown of *KRAS* and *PIK3CA* in MCF-10A DKI cells.
- Figure S3: Inhibition of KRAS or PI3K signaling limits anchorage-independent growth of MCF-10A DKI cells.
- Figure S4: Inhibition of MEK by AZD6244 does not impact cell viability.
- Figure S5: Large CNV determination in MCF-10A cells by SNP analysis after whole genome sequencing.
- Figure S6: BRD9 transcript is downregulated after CRISPR inactivation of *KRAS* and *PIK3CA*.
- Figure S7: CRISPR/Cas9 knockdown of BRD9 inhibits MYC and blocks anchorage-independent growth.
- Figure S8: Small molecule inhibition of BRD9 has no effect on migration capacity.
- Figure S9: BRG1 co-immunoprecipitates with endogenous BRD9.
- Figure S10: MYC and BRD9 are upregulated in MCF-10A DKI cells.

- Table S1: Top ranked Hallmark gene signatures for MCF-10A mutant vs. WT determined by GSEA.
- Table S2: Top 30 DE genes in mutant vs. WT cells.
- Table S3: Single guide RNA oligo sequences
- Table S4: PCR primer sequences

Supplementary experimental procedures

Whole Genome Sequencing

High molecular weight genomic DNA was prepared from MCF-10A cells using Ultra-Pure phenol:chloroform:isoamyl alcohol (ThermoFisher). Purity, quantity and size of DNA were assessed by NanoDrop and agarose gel electrophoresis. DNA was sequenced by GeneWiz on an Illumina Novaseq. Raw sequencing data were processed into fastq files using CASAVA 1.8 (Illumina). The raw reads used for variant calling are available from the National Center for Biotechnology Information Sequence Read Archive (NCBI SRA) as Project PRJNA526932. The resulting reads were aligned to HG38 using Burrows–Wheeler Aligner version 0.7.10 [55].

Copy Number Variation Analysis

Whole genome sequencing data were used to identify regions of the genome with significant copy number variations (CNVs). The aligned sequences were analyzed using ExomeDepth version 1.0.5 [56] in R. The regions with a Bayes factor of >20 were selected as significant and annotated with the Conrad's frequent CNV regions and overlapping genes [57].

For large regions, variant calls were used to determine CNV by changes in SNP allele frequency from the whole genome sequencing data. The allele frequencies present in wild type and mutant MCF-10A cell lines were plotted against genomic coordinates in a way analogous to the methods used for CNV determination by SNP microarray [58]. Regions of copy number gain are visible as changes in heterozygous allele frequency from 1/2–1/3, 2/3, 1/4, 3/4, etc.

Immunoblotting

Primary antibodies used for supplementary data: anti-phospho-AKT (T308) (CST #9272), anti-phospho-p70S6K1 (CST #9205), anti-p70S6K1 (CST #9202), Ras-G12V (CST #1441), anti-BIG-H3 (TGFBI) (CST #2719), anti-β actin (CST #4970) (Cell Signaling Technology), anti-LPCAT1 (Proteintech #66044), anti-TRIP13 (Bethyl Laboratories #A303-606A) and anti-MISU (NSUN2) (Millipore #ABE1076).

Growth on a non-binding surface

To assess anchorage-independent growth, 96-well plates were coated with 20 mg/ml poly2-hydroxymethacrylate (polyHEMA) (Sigma) in 95% ethanol. Plates were dried overnight in a non-humidified 37°C incubator prior to UV sterilization for 30 min. Exponentially growing cells were resuspended at 5x104 cells/ml complete EGF-free growth media, and 200 µl were plated per well to yield 10,000 cells per well. Cell viability was quantified after 72 h by addition of Resazurin dye (Sigma) and absorbance at 595 nm on a plate reader.

CRISPR/Cas9

Initial experiments to knock out PIK3CA or KRAS were performed using pSpCas9(BB)-2A-GFP (PX458) (provided by Feng Zhang, Addgene plasmid #48138). Six 20-nucleotide guide sequences, including a 5′ PAM, were designed to target open reading frames of each gene, using the CRISPR Design Tool (http://tools.genome-engineering.org). Oligos with 40 nt homology arms (Integrated DNA Technologies) were phosphorylated, annealed and ligated into PX458 for co-expression with Cas9. Plasmids were transfected into MCF-10A cells using Lipofectamine 3000 (ThermoFisher) and FACS-sorted for GFP expression. Guide RNA validation was evaluated by High-Resolution Melting on a Roche LightCycler 96 Instrument, comparing PCR-amplified DNA from transfected and non-transfected cells using PIK3CA and KRAS exon-specific primers.

PCR amplification of KRAB

DNA was isolated from DKI cells expressing dCas9/KRAB and sgRNA targeting KRAS or PIK3CA using a DNeasy Blood and Tissue kit (Qiagen). KRAB was amplified using gene-specific primers and the Expand HiFi PCR system (Roche) prior to resolution on a 1% agarose gel.

Cell viability assay and cell cycle analysis

For detection of cell death, cells were stained with propidium iodide (PI) and annexin V-conjugated to Alexa Fluor™ 488 according to the manufacturer's instruction (dead cell apoptosis kit, Invitrogen). Immediately after staining, the cells were analyzed on a flow cytometer using 488-nm excitation for Alexa Fluor™ 488 and a 640 nm filter for PI detection. 10,000 cells were acquired in an LSR II flow cytometer using FACS Diva 6 software (BD Biosciences). Percentages of dead cells were determined by dual-color analysis.

For cell cycle analysis, cells were removed from plates using 0.25% Trypsin and resuspended into EBSS. Cells were fixed by dropping into 70% ethanol solution. The fixed cells were centrifuged and resuspended in 1 mL of PBS containing 0.1% Triton X-100. RNA was removed by treatment with 50 μg RNAse A. Cells were stained with 20 μg/mL Propidium Iodide. Stained cells were analyzed on an LSR II flow cytometer using FACS Diva 6 software (BD Biosciences). Cell cycle analysis was performed using FlowJo v 10.1 using the Watson model [59].

IC50 calculations

In order to understand the potency of the inhibitors A66 (Tocris), AZD6244 (Selleckchem), I-BRD9 (Tocris) and KJ-Pyr-9 in MCF-10A cells, dose-response curves were generated in GraphPad Prism v. 6.0. Exponentially growing cells were seeded into 96-well plates in complete media at a density of 1500 cells/well. The media was exchanged 24 h later for EGF-free media containing inhibitor in log-fold increases in concentration. After 72 h, cells were stained with 10 μg/ml Resazurin (Sigma) and viability assessed by quantifying the fluorescence at 595 nm on a plate reader. This data was fit to a curve using nonlinear regression, log (inhibitor) vs. response, variable slope (four parameters). Here, the relative IC50 is defined as the concentration that defines the point halfway between the top (no inhibitor) and bottom plateaus (maximum cell death).

References:

55. Li H, Durbin R. Fast and accurate long-read alignment with Burrows-Wheeler transform. *Bioinformatics* **2010**, 26(5), 589-95. DOI: 10.1093/bioinformatics/btp698.
56. Plagnol V, Curtis J, Epstein M, Mok KY, Stebbings E, Grigoriadou S, et al. A robust model for read count data in exome sequencing experiments and implications for copy number variant calling. *Bioinformatics* **2012**, 28(21), 2747-54. DOI: 10.1093/bioinformatics/bts526.
57. Conrad DF, Pinto D, Redon R, Feuk L, Gokcumen O, Zhang Y, et al. Origins and functional impact of copy number variation in the human genome. *Nature* **2010**, 464(7289), 704-12. DOI: 10.1038/nature08516.
58. Wang K, Bucan M. Copy Number Variation Detection via High-Density SNP Genotyping. *CSH Protoc* 2008, **2008**, pdb top46. DOI: 10.1101/pdb.top46.
59. Watson JV, Chambers SH, Smith PJ. A pragmatic approach to the analysis of DNA histograms with a definable G1 peak. *Cytometry* **1987**, 8(1), 1-8. DOI: 10.1002/cyto.990080101.

Figures

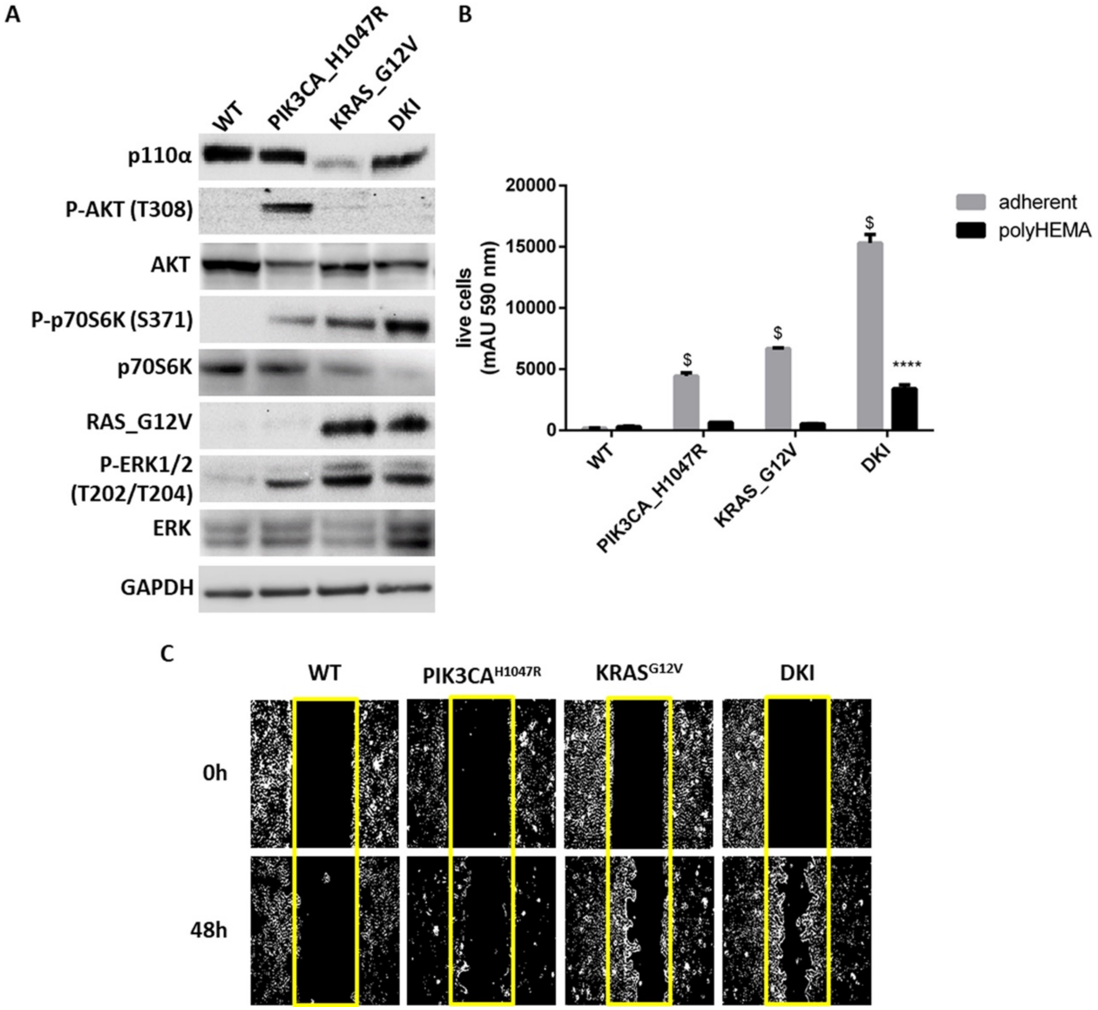

Figure S1: DKI cells have activated PI3K and KRAS pathways and show synergistic growth effects. (A) MCF-10A cells were grown in EGF-free media to subconfluence prior to immunoblotting using antibodies that characterize PI3K and KRAS activation. (B) MCF-10A cells were grown in EGF-free media for 72h on tissue culture-coated or polyHEMA-coated 96-well plates and stained using Alamar blue. $ and ****, *p* = significant by 2-way ANOVA using Tukey's multiple comparison test (α = 0.05. Mean ± SEM, n = 3). (C) DKI cells migrate faster across a scratched surface. Near-confluent MCF-10A cell monolayers were scratched and allowed to grow for 48h in EGF-free media prior to imaging by light microscope at 50x magnification.

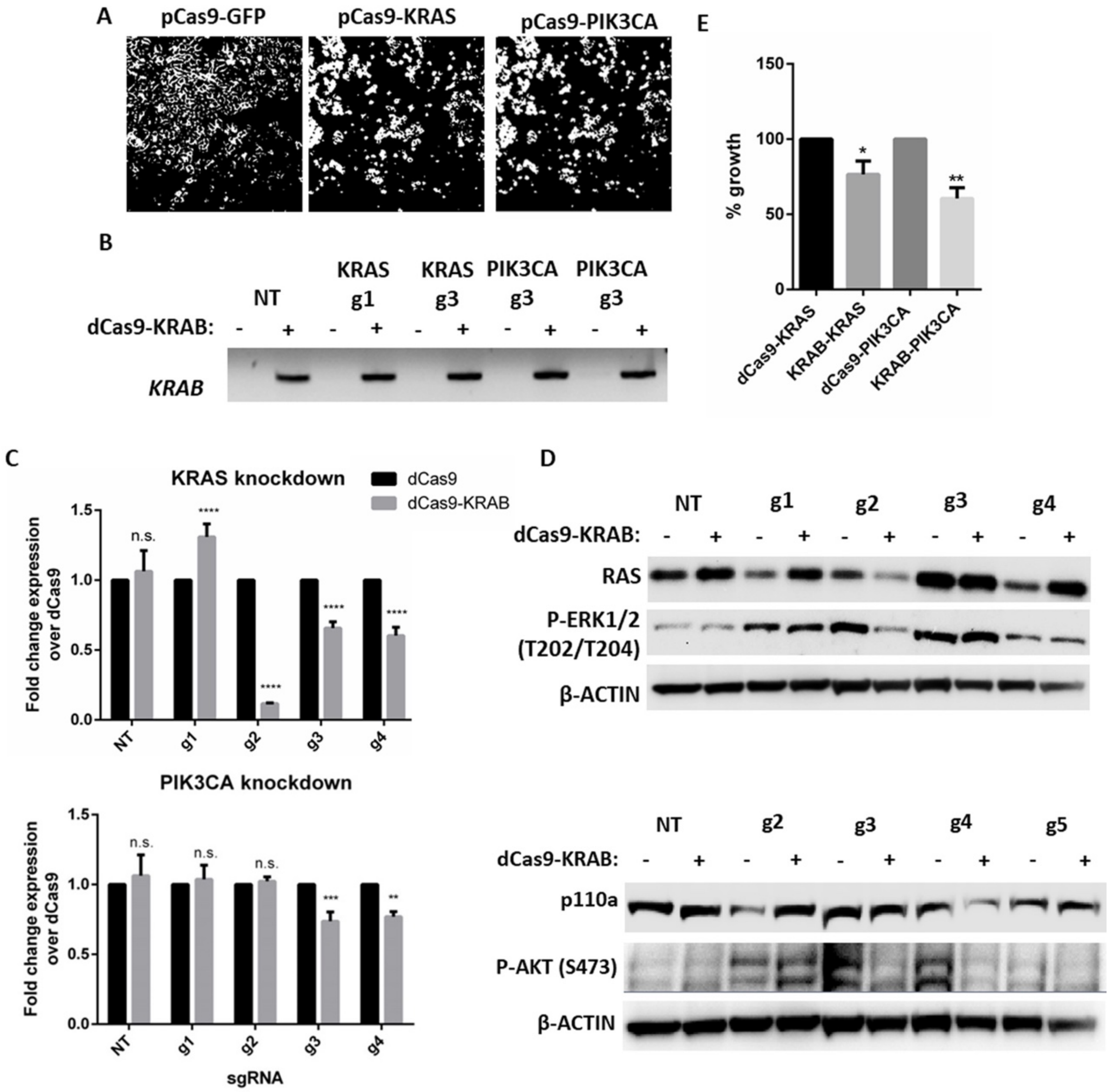

Figure S2: CRISPR/Cas9-mediated knockdown of *KRAS* and *PIK3CA* in MCF-10A DKI cells. (A) DKI cell death 72 h after transfection of PX458 encoding Cas9 and sgRNAs targeting an exon of *KRAS* or *PIK3CA*. Images captured by light microscope at 50x magnification. (B) Agarose gel showing PCR amplification of *KRAB* from DKI cells stably expressing dcas9-KRAB and sgRNA. (C) RT-qPCR data from knockdown of *KRAS* or *PIK3CA* by lentiviral expression of dCas9-KRAB and sgRNAs (g1-g5) in DKI cells. Relative quantitation based on Livak's ΔΔCt with *RPLPO* as the calibrator gene. (D) Western blots showing corresponding downregulation of KRAS and p110α. (E) Stably transformed DKI cells expressing dCas9-KRAB with sgRNAs targeting *KRAS* or *PIK3CA* were grown in EGF-free media for 72 h. Live cells were stained using Alamar blue. *, p = significant by 2-way ANOVA, Sidak's multiple comparisons test (α=0.05. Mean ± SEM, n = 3). n.s. = not significant. NT = non-targeting sgRNA.

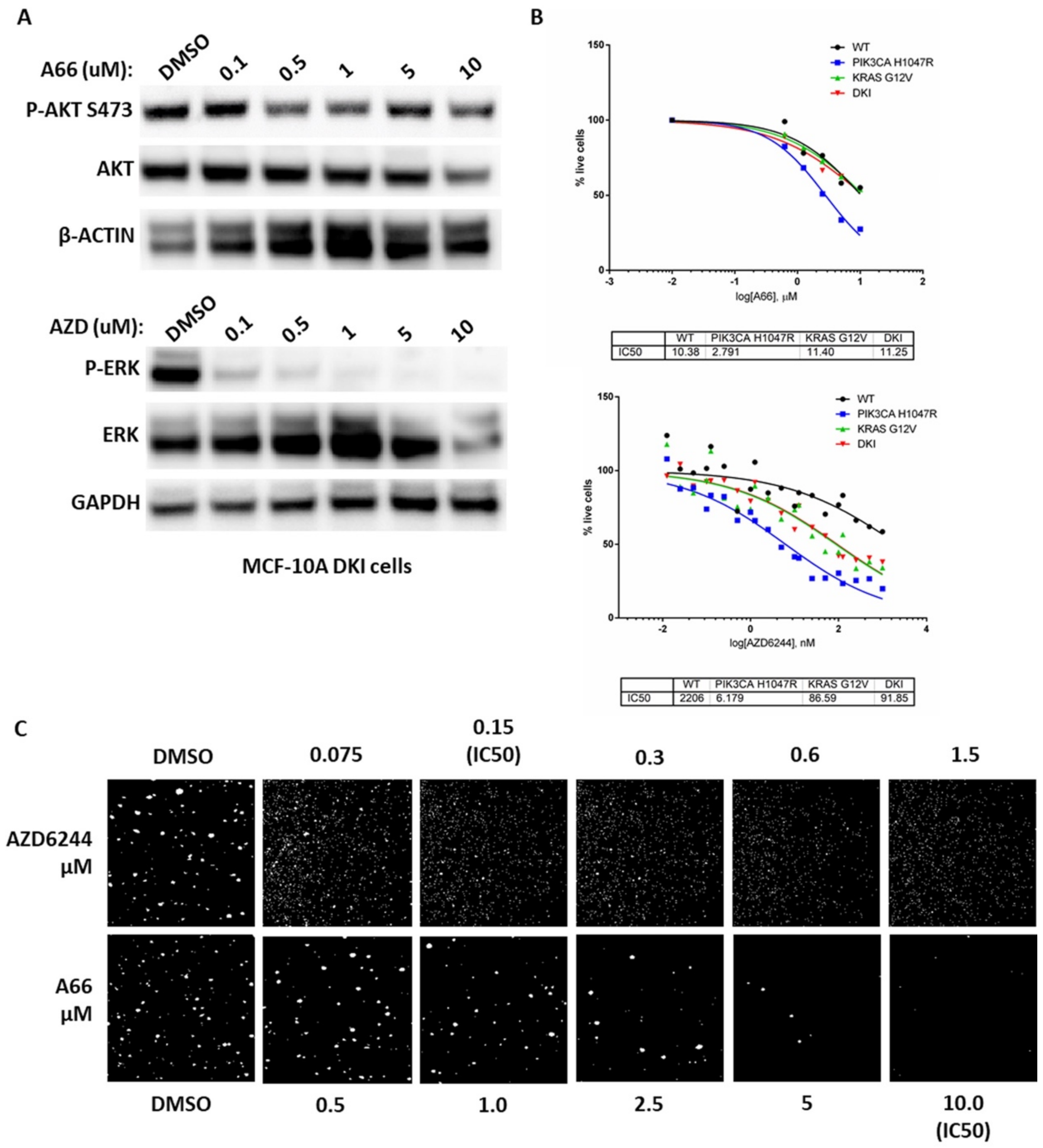

Figure S3: Inhibition of KRAS or PI3K signaling limits anchorage-independent growth of MCF-10A DKI cells. (A) Immunoblots showing p110*α* and KRAS signaling in DKI cells in response to increasing doses of p110*α* inhibitor, A66, or MEK inhibitor, AZD6244, for 24 h. (B) Inhibitory action of A66 and AZD6244 in MCF-10A cells calculated 72 h after treatment by measuring cell viability using Alamar blue. IC50 values calculated from non-linear regression curves, log[antagonist] vs. normalized response, in GraphPad Prism v.6. (C) Anchorage-independent growth of DKI cells in 0.3% agar after treatment with A66 or AZD6244 for 21 days. Images captured by light microscope at 10x magnification.

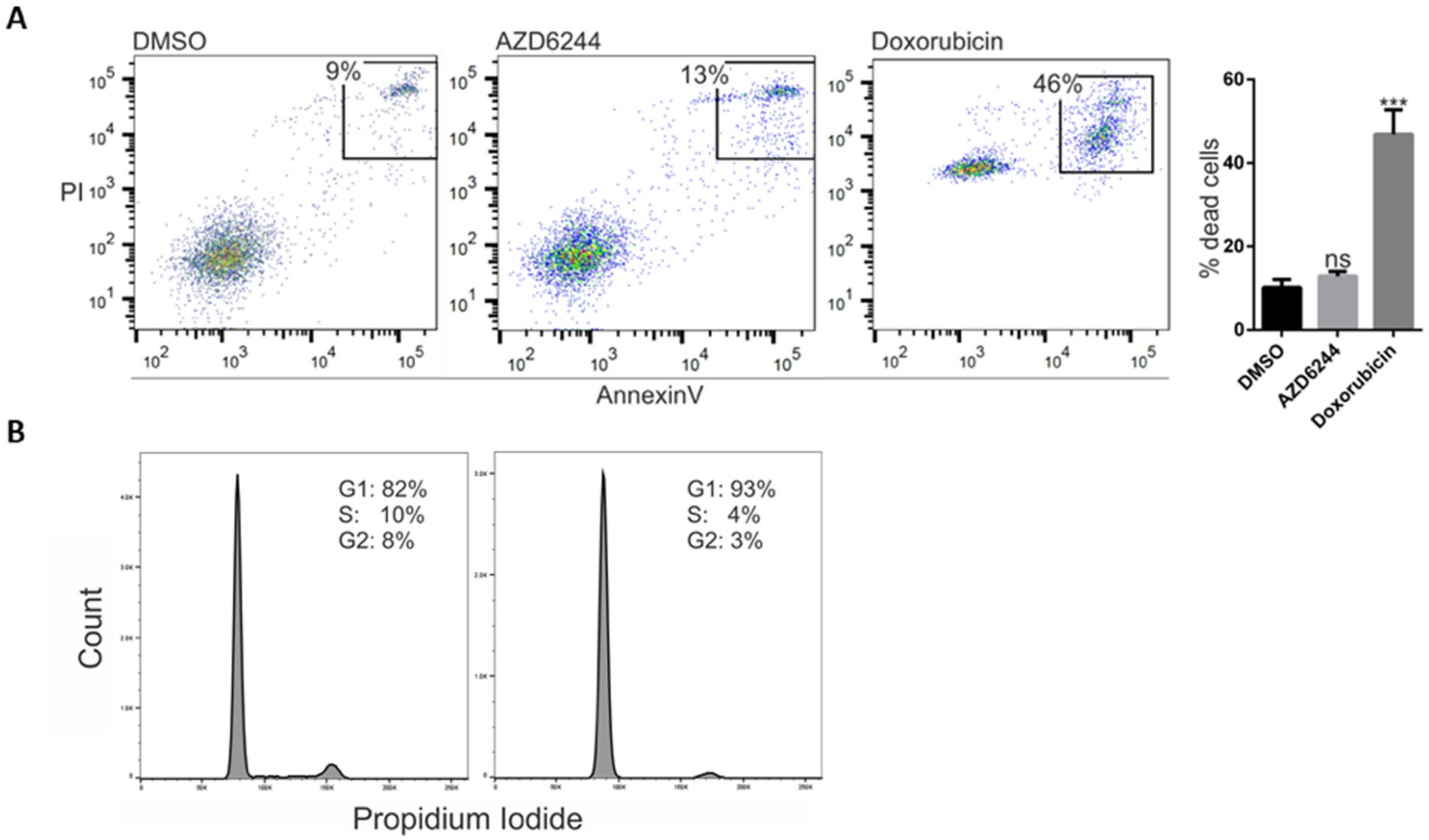

Figure S4: Inhibition of MEK by AZD6244 does not impact cell viability. (A) DKI cells were treated with vehicle (DMSO) or 200 nM AZD6244 for 48h. Doxorubicin at 1 μM was included as a positive control. Cells were stained with annexin V and propidium iodide (PI) and subjected to flow cytometry. Bar graph shows quantitation of cell death (annexin V+/PI+ population); error bars indicate SD of three replicates, ***$p < 0.001$ and ns denotes not significant (unpaired t-test). (B) PI histograms were used to analyze the effect of AZD6244 on cell cycle. The percentage of cells in each phase (G1, S, and G2) is shown. No sub-G1 peak was observed.

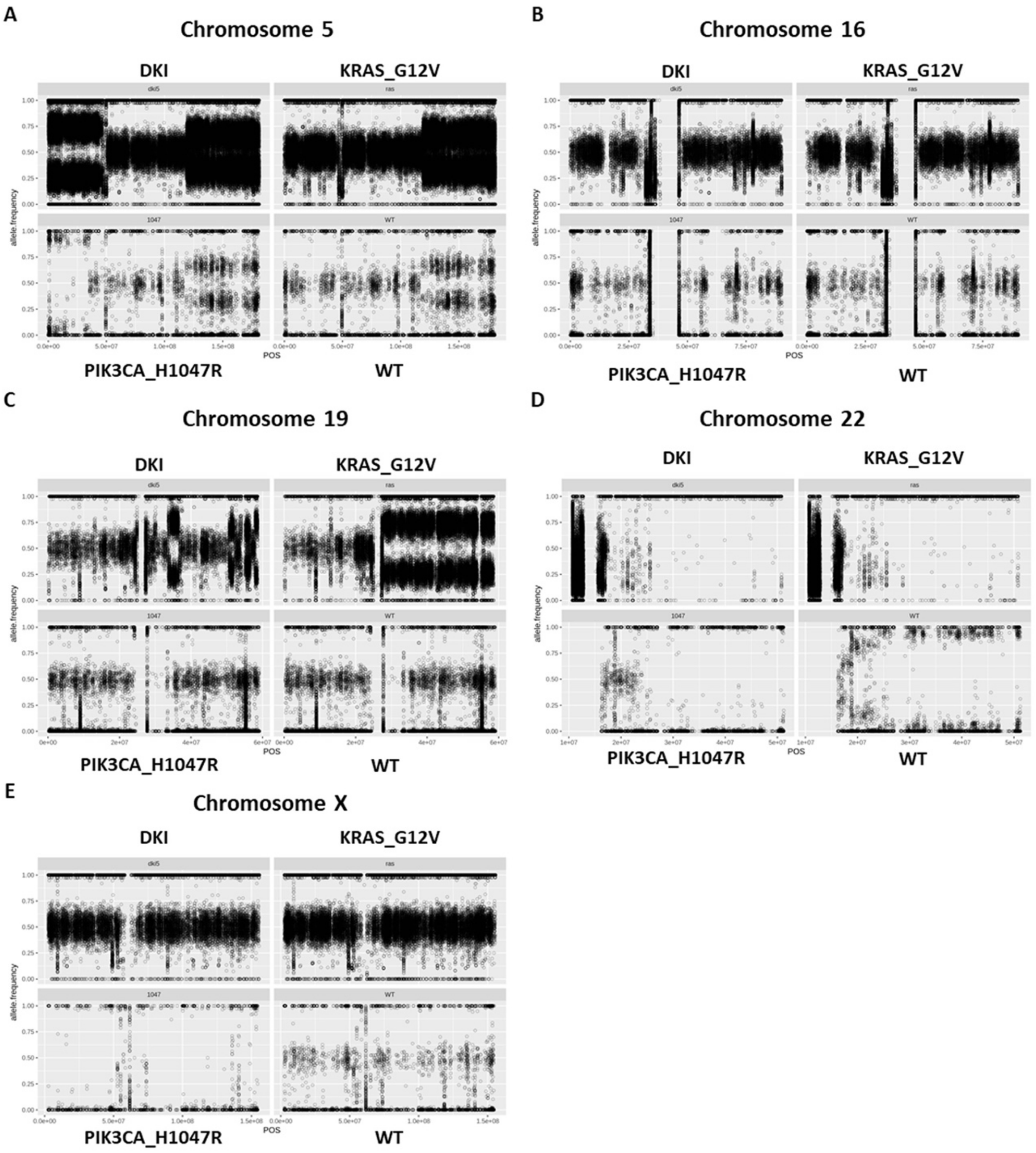

Figure S5: Large CNV determination in MCF-10A cells by SNP analysis after whole genome sequencing. Frequency plots show allele frequency vs. base pair position (POS) on selected chromosomes in each cell line.

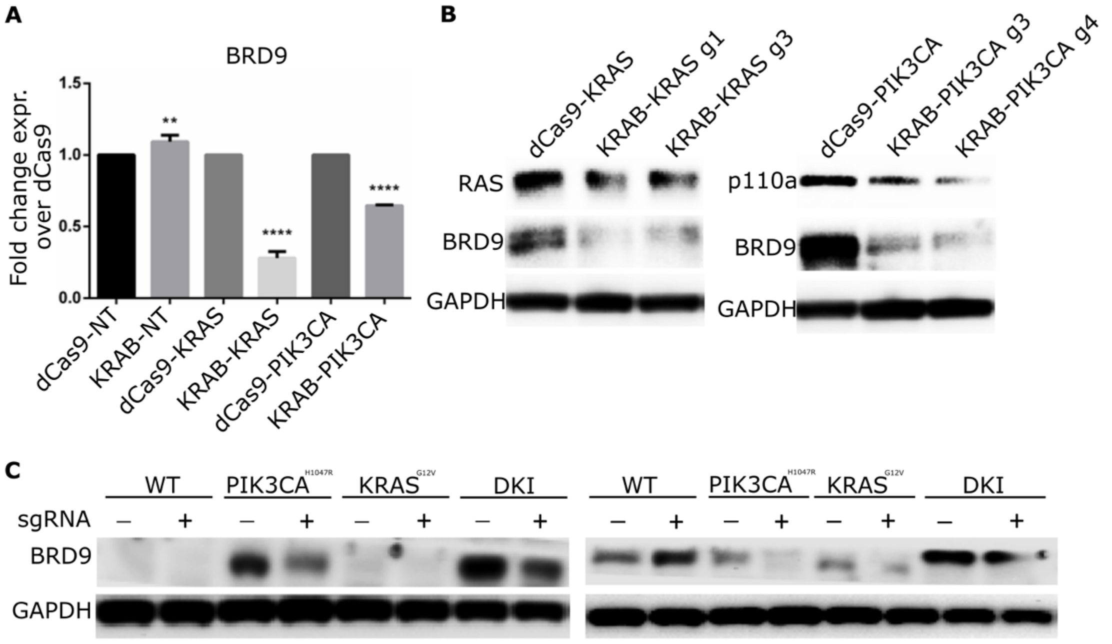

Figure S6: BRD9 transcript is downregulated after CRISPR inactivation of *KRAS* and *PIK3CA*. (A) RT-qPCR data showing *BRD9* mRNA in DKI cells expressing lentiviral CRISPR/dCas9-sgRNA constructs. Data shows relative quantitation (Livak's ΔΔCt) with *RPLPO* as the calibrator gene. *, p = significant by one-way ANOVA with Tukey's multiple comparisons test (Mean ± SEM, n = 3). Immunoblots showing (B) p110α, RAS and BRD9 protein in DKI cells and (C) a comparison across all MCF-10A cell lines expressing lentiviral CRISPR/dCas9-sgRNA constructs.

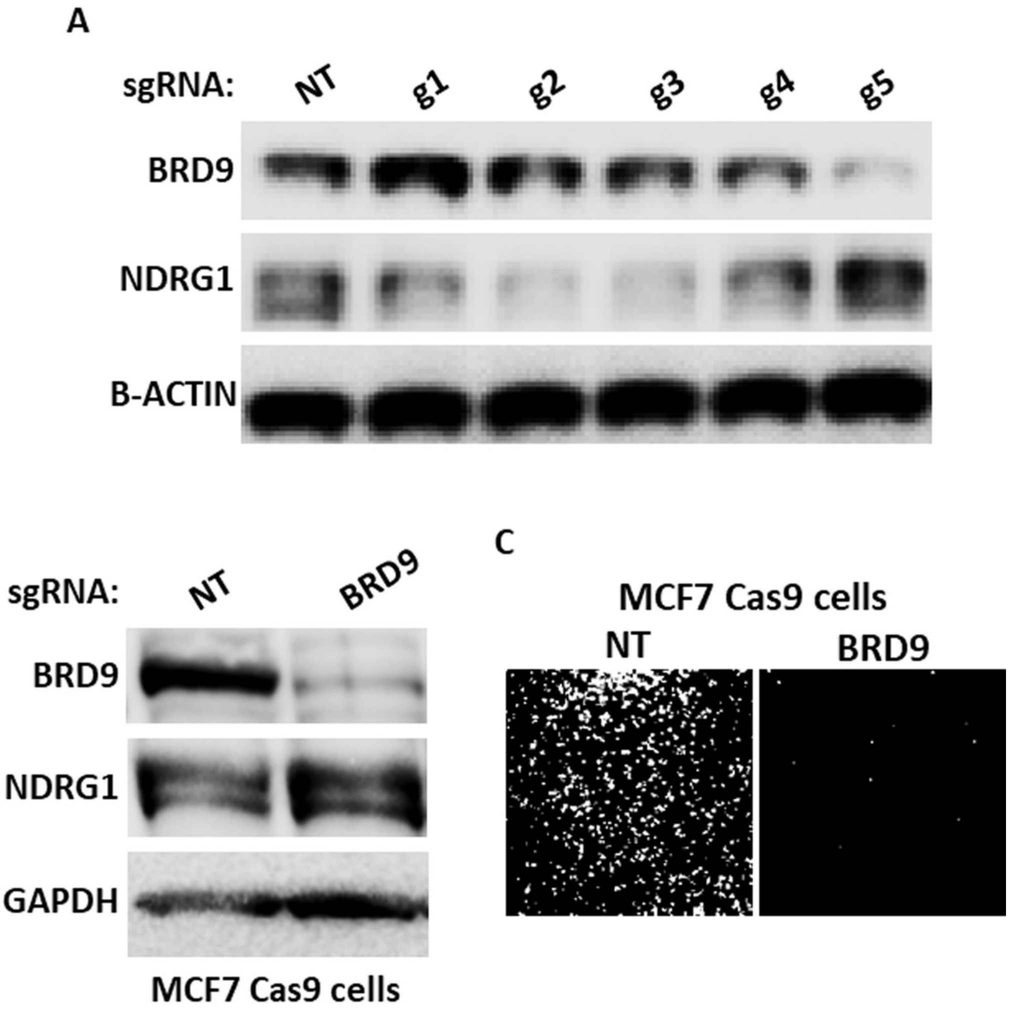

Figure S7: CRISPR/Cas9 knockdown of BRD9 inhibits MYC and blocks anchorage-independent growth. (A) Immunoblots showing sgRNA (g1-5) knockdown efficiency against *BRD9* exon regions in DKI cell expressing Cas9. (B) BRD9 protein levels in MCF7 cells expressing lentiviral Cas9 and *BRD*9-targeting sgRNA. (C) Soft agar growth of MCF7 cells expressing Cas9 and *BRD*9-targeting sgRNA. Images captured by light microscope at 10x magnification.

A

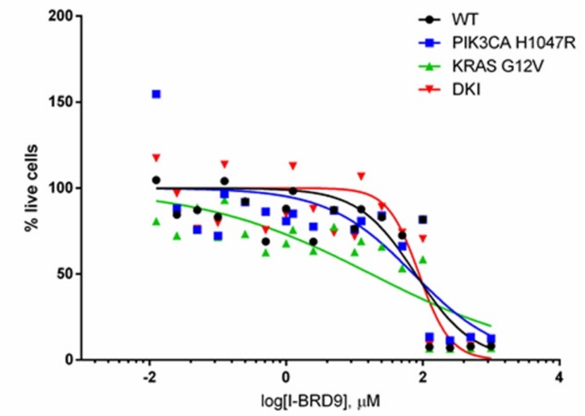

	WT	PIK3CA H1047R	KRAS G12V	DKI
IC50	78.41	73.22	17.50	88.00

B

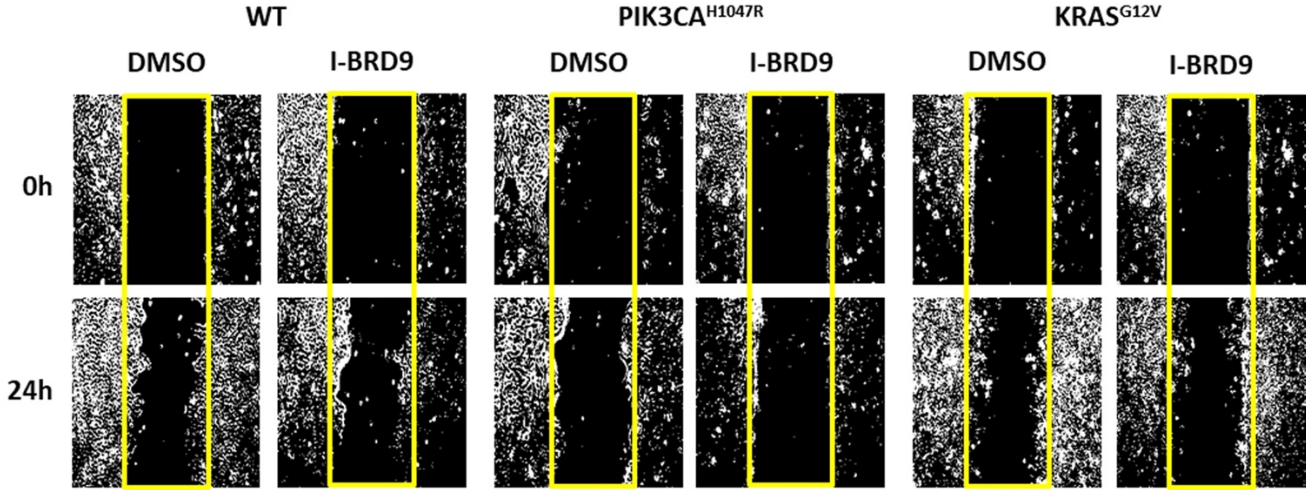

Figure S8: Small molecule inhibition of BRD9 has no effect on migration capacity. (A) Inhibitory action of I-BRD9 in MCF-10A cells calculated 72 h after treatment by measuring cell viability using Alamar blue. IC50 values calculated from non-linear regression curves, log[antagonist] vs. normalized response, in GraphPad Prism v.6. (B) Migration of MCF-10A WT and single mutant cells across a scratched wound over 48 h after treatment with IC50 (μM) concentrations of I-BRD9. Images captured by light microscope at 50x magnification.

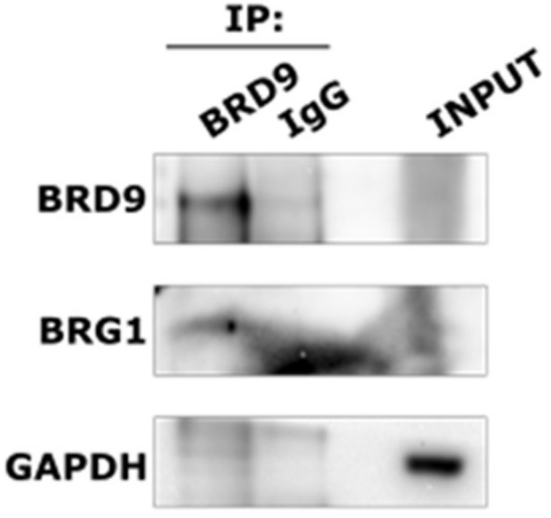

Figure S9: BRG1 co-immunoprecipitates with endogenous BRD9. Immunoblots showing Co-IP of BRG1 with BRD9 from MCF-10A DKI nuclear extracts.

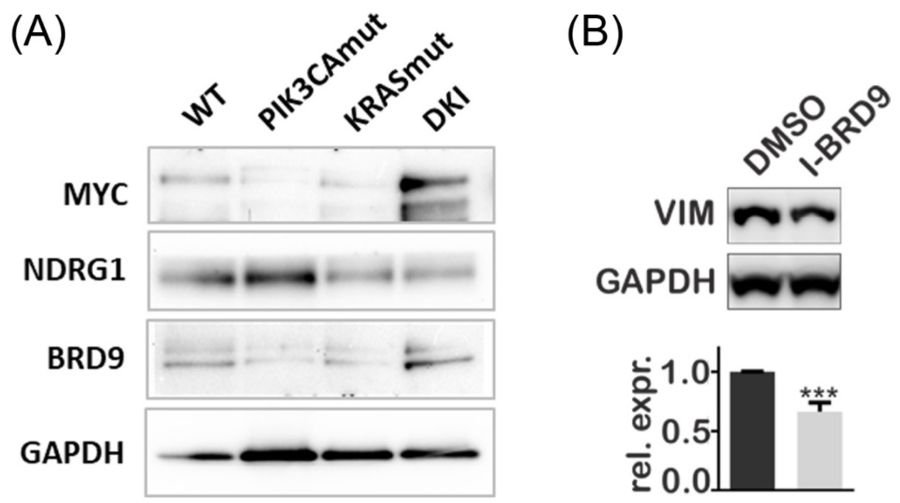

Figure S10: MYC and BRD9 are upregulated in MCF-10A DKI cells. (A) MCF-10A cells were grown in EGF-free media for 24 h prior to SDS-PAGE and immunoblotting for BRD9, MYC and negatively regulated MYC target, NDRG1. (B) Protein levels of VIM in DKI cells following 48 h treatment with 20 μM I-BRD9. Bar graph shows relative density of VIM protein normalized to GAPDH. Error bars indicate SD of 4 replicates, ***p<0.001 (unpaired t-test).

Tables

Table S1: Top ranked Hallmark gene signatures for MCF-10A mutant vs. WT determined by GSEA.

Rank	DKI vs. WT	PIK3CA_H1047R vs. WT	KRAS_G12V vs. WT
1	EPITHELIAL_MESENCHYMALT RANSITION	TNFA_SIGNALING_VIA_ NFKB	KRAS_SIGNALING_DN
2	COAGULATION	CHOLESTEROL_ HOMEOSTASIS	TNFA_SIGNALING_VIA_ NFKB
3	E2F_TARGETS	INFLAMMATORY_RESPONSE	EPITHELIAL_MESENCHYMALT RANSITION
4	G2M_CHECKPOINT	IL6_JAK_STAT3_SIGNALING	APICAL_JUNCTION
5	KRAS_SIGNALING_UP	ANDROGEN_RESPONSE	E2F_TARGETS
6	APICAL_JUNCTION	UNFOLDED_PROTEIN_ RESPONSE	TGF_BETA_SIGNALING
7	TNFA_SIGNALING_VIA_ NFKB	MYC_TARGETS_V2	IL2_STAT5_SIGNALING
8	INFLAMMATORY_RESPONSE	MTORC1_SIGNALING	KRAS_SIGNALING_UP
9	COMPLEMENT	P53_PATHWAY	G2M_CHECKPOINT
10	MTORC1_SIGNALING	APICAL_JUNCTION	ANDROGEN_RESPONSE
11	UV_RESPONSE_UP	WNT_BETA_CATENIN_ SIGNALING	WNT_BETA_CATENIN_ SIGNALING
12	TGF_BETA_SIGNALING	COMPLEMENT	ANGIOGENESIS
13	WNT_BETA_CATENIN_ SIGNALING	HYPOXIA	COMPLEMENT
14	ANDROGEN_RESPONSE	INTERFERON_GAMMA_ RESPONSE	INFLAMMATORY_RESPONSE
15	IL2_STAT5_SIGNALING	PI3K_AKT_MTOR_ SIGNALING	ESTROGEN_RESPONSE_LATE
16	SPERMATOGENESIS	IL2_STAT5_SIGNALING	COAGULATION
17	HEDGEHOG_SIGNALING	APOPTOSIS	IL6_JAK_STAT3_SIGNALING
18	MITOTIC_SPINDLE	ALLOGRAFT_REJECTION	UV_RESPONSE_DN
19	PI3K_AKT_MTOR_ SIGNALING	HEDGEHOG_SIGNALING	HYPOXIA
20	KRAS_SIGNALING_DN	KRAS_SIGNALING_UP	MTORC1_SIGNALING

Table S2: Top 30 DE genes in mutant vs. WT cells.

Rank	DKI vs WT	Log2FC	H10 vs WT	Log2FC	KRAS vs WT	Log2FC
1	COL4A1	4.19	IL1A	3.58	COL4A1	3.93
2	TFPI2	4.09	PI3	7.52	TGFBI	4.61
3	FAM110C	4.47	A2ML1	5.13	ADAM19	4.62
4	ARL4C	3.84	KRT6B	4.89	ARL4C	3.41
5	TCF19	3.73	PTPRZ1	2.85	DHRS3	4.87
6	DHRS3	5.33	SPRR1B	4.84	LTBP1	3.10
7	TINAGL1	4.18	FGFBP1	3.10	LMNB2	2.85
8	LMNB2	3.43	KRT14	3.45	TCF19	3.18
9	PROSER2	3.42	NRP2	1.92	TFPI2	3.03
10	ADAM19	4.66	NAV3	2.59	JAG1	2.93
11	PHLDA1	3.40	ARL4C	1.72	PROSER2	3.02
12	TGFBI	4.23	PHLDA1	1.41	TINAGL1	3.37
13	CCT2	2.80	PNLIPRP3	2.09	PHLDA1	2.74
14	TXNRD1	2.91	EPGN	2.27	ZBED2	2.76
15	UBA2	3.25	SLPI	2.04	FAM110C	3.29
16	TRIP13	3.44	WNT7A	1.62	CCT2	2.29
17	LTBP1	3.06	NCF2	2.95	TONSL	2.66
18	NSUN2	3.04	SERPINB1	1.21	ARNTL	2.75
19	MCM2	3.61	TNFAIP3	3.31	UHRF1	3.00
20	UHRF1	3.52	IRS1	1.75	CARD10	3.16
21	ANXA5	2.36	GALNT6	1.16	MAP3K9	2.48
22	CARD10	3.73	TGFA	1.23	MB21D1	3.00
23	GSR	2.82	XDH	1.30	IL1A	3.62
24	COL16A1	3.81	S100A9	3.65	P3H2	3.01
25	TONSL	3.03	GJB2	1.83	MYBL2	3.04
26	MYBL2	3.75	C1orf116	1.32	FAM83A	2.88
27	ARNTL	3.07	AKR1C1	2.10	MBOAT2	2.25
28	CDC6	2.79	ZBED2	1.24	FRMD6	2.32
29	FAM129B	2.89	IER3	1.30	COL4A2	2.47
30	U2AF2	2.24	IL1RN	2.11	CDC6	2.39

Fold change (Log2FC) sorted by P value.

Table S3: Single guide RNA oligo sequences

Target	guide name	genomic sequence	Orien-tation	forward oligo	reverse oligo
**KRAS_TSS**	g1	GTCTGAAGAA GAATCGAGCG	antisense	caccgCGCTCGATTC TTCTTCAGAC	aaacGTCTGAAGA AGAATCGAGCGc
	g2	CTAATTCATTC ACTCGCCGC	sense	caccgCTAATTCATT CACTCGCCGC	aaacGCGGCGAGT GAATGAATTAGc
	g3	GCTCCCAGTC CGAAATGGCG	sense	caccgGCTCCCAGTC CGAAATGGCG	aaacCGCCATTTCG GACTGGGAGCc
	g4	GAGGGACTGC CGGACCCACG	sense	caccgGAGGGACTG CCGGACCCACG	aaacCGTGGGTCC GGCAGTCCCTCc
	g5	CGTACCTCTCT CCCGCACCT	sense	caccgCGTACCTCTC TCCCGCACCT	aaacAGGTGCGGG AGAGAGGTACGc
**PIK3CA_ TSS**	g1	AAGAAGCGGA AGCGAAATTG	antisense	caccgCAATTTCGCT TCCGCTTCTT	aaacAAGAAGCGG AAGCGAAATTGc
	g2	AACTGTACAT AAACTTCGGG	sense	caccgAACTGTACAT AAACTTCGGG	aaacCCCGAAGTTT ATGTACAGTTc
	g3	ACCCTCACTA CTGCAGAAGA	sense	caccgACCCTCACTA CTGCAGAAGA	aaacTCTTCTGCAG TAGTGAGGGTc
	g4	TACTACACGT ACGCTGTCCT	sense	caccgTACTACACGT ACGCTGTCCT	aaacAGGACAGCG TACGTGTAGTAc
	g5	GGGCAGAGCC TACAATCCCC	antisense	caccgGGGGATTGT AGGCTCTGCCC	aaacGGGCAGAGC CTACAATCCCCc
**BRD9**	g1	AGATACCGTG TACTACAAGT	sense	caccgAGATACCGT GTACTACAAGT	aaacACTTGTAGTA CACGGTATCTc
	g2	AGGGAGCACT GTGACACGGA	sense	caccgAGGGAGCAC TGTGACACGGA	aaacTCCGTGTCAC AGTGCTCCCTc
	g3	CTTGACGGAC AGTACCGCAG	sense	caccgCTTGACGGAC AGTACCGCAG	aaacCTGCGGTACT GTCCGTCAAGc
	g4	ACTCCAGTTA CTATGATGAC	sense	caccgACTCCAGTTA CTATGATGAC	aaacGTCATCATAG TAACTGGAGTc
	g5	TCCAGATGGG CTATCTGAAG	sense	caccgTCCAGATGG GCTATCTGAAG	aaacCTTCAGATAG CCCATCTGGAc
	g6	CAGGAAAAGC AAAAAATCCA	antisense	caccgTGGATTTTTT GCTTTTCCTG	aaacCAGGAAAAG CAAAAAATCCAc
**Non-targeting**	NT	TGCCCACTTAGCAACACTCT			

Table S4: PCR primer sequences

Application	Target	Sense primer	Antisense primer
**sequencing**	KRAS	TGCTGAAAATGACTGAATATAAACTTGTG	GGTCCTGCACCAGTAATATGCA
	PIK3CA	CATTTGCTCCAAACTGACCA	TGAGCTTTCATTTTCTCAGTTATCTTTTC
	dCas9		CCAAAGAGGTGCTGGACG
**PCR**	KRAS_G12V (Exon 2)	CGATACACGTCTGCAGTCAAC	ACCCTCTCACGAAACTCTGA
	PIK3CA_H1047R (Exon 20)	CATCATTTGCTCCAAACTGACCA	CCTATGCAATCGGTCTTTGCC
**RT-qPCR**	TRIP13	TGCTGATTGATGAGGTGGAGAG	GGTTGCACAAGTATCACGCA
	VIM	CGGGAGAAATTGCAGGAGGA	AAGGTCAAGACGTGCCAGAG
	BRD9	GATGAAGCCAAGGTTGGGGA	GGTCCAGCTCCTTCTTCACC
	PEAR1	GCCAGAGATCCTGTCAGCC	TCTGGGCACAGTTTTCTCCC
	LPCAT1	TGCACGAGCTGCGCC	CACAACCTTCCTCCACAGGG
	CLPTM1L	ACCGGGGAGTCTGATACACA	CCCCAGCTGGATCATCTTCA
	NSUN2	ACAGCCACTGAGTTGGTATCC	TTATGATGAGGCCGCACGTT
	TGFBI	TAACGGCCAGTACACGCTTT	GTTCAGCAGGTCTCTCAGGG
	FAM110C	CTGATAGAGCAGGTGCCCAG	AAGGTTTGCTTCCTCGGGTC
	TFPI2	CAGGAGCCAACAGACTACGG	AAATTGTTGGCGTTGCCCTC
	COL4A1	TTTGAAAAAGCAATGGCACTCC	GACCCCCGGGAGAAATAGGT
	KRAS	CGTAGGCAAGAGTGCCTTGA	GGTCCTGCACCAGTAATATGC
	PIK3CA	CATCATTTGCTCCAAACTGACCA	CCTATGCAATCGGTCTTTGCC
	MYC	GCAAAAGCTCATTTCTGAAGAGG	TTCATAGGTGATTGCTCAGGAC
	RPLP0	GAAACTCTGCATTCTCGCTTC	GGTGTAATCCGTCTCCACAG
**ChIP PCR**	MYC_E1	CCAGTCCCAGCTAATGGATAAA	TGCACTGTCTCTAGATTGTCTTC
	MYC_E2	TCTTTCCAGAGCAGCATTCC	AGGCAGTAGATGGCAGTAGA
	VIM_TSS	TGCAGTTCGCATTTCCTCCT	AGGACTGGCTCTCATTGTGC
	BRD4_EXON10	ACGTATGAGTCGGAGGAAGA	GGGAGCTTGTTGATGTCCA
	SUMO2_TSS	CCTTCGTGGTGGAGCATAAT	GGCCTATGGCTTACGTCTAAAG
	NEG	GGTCAGGCCAACTTGATTGT	AATTTGTGTTGGGCCACATT
